# Supplementary material for: New Benzimidazoles Targeting Breast Cancer: Synthesis, Pin1 Inhibition, 2D NMR Binding, and Computational Studies
Source: Molecules. 2022 Aug 17;27(16):5245. doi: 10.3390/molecules27165245 (PMC9414874; doi:10.3390/molecules27165245)

New Benzimidazoles Targeting Breast Cancer: Synthesis, Pin1 Inhibition, 2D NMR Binding, and Computational Studies

Samira Nashaat [1], Morkos A. Henen [1,2,*], Shahenda M. El-Messery [1,*] and Hassan Eisa [1]

1 Department of Pharmaceutical Organic Chemistry, Faculty of Pharmacy, Mansoura University, Mansoura P.O. Box 35516, Egypt; samiranashaat2@gmail.com (S.N.); nmr_afpnoesy@outlook.com (H.E.)
2 Department of Biochemistry and Molecular Genetics, University of Colorado Denver, Denver, CO 80204, USA
* Correspondence: morkos.henen@ucdenver.edu (M.A.H.); selmessery@gmail.com (S.M.E.-M.); Tel./Fax: +20-50-2200242 (S.M.E.-M.)

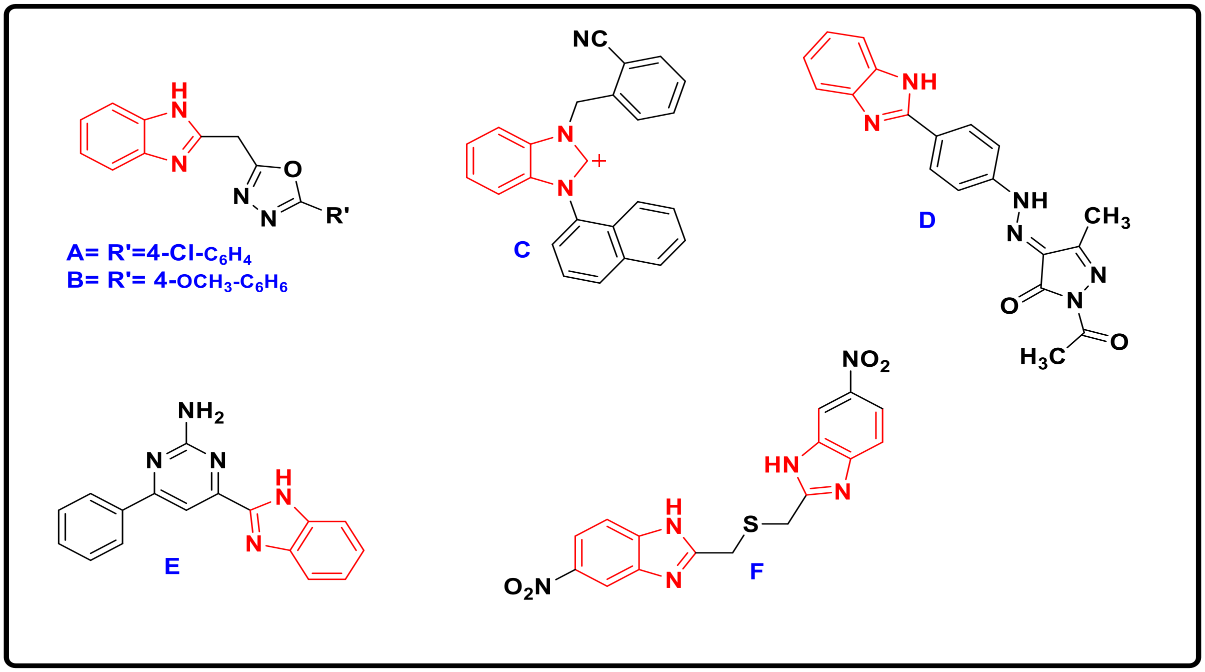

Chart S1. Structures of some known benzimidazoles derivatives with antiproliferative activity against breast cancer cell lines with IC_{50} against MCF7 is >100μM.

List of spectroscopic spectra of the compounds

compound	^{1}H NMR	^{13}C NMR	IR
**4**	δ: 3.05 (s, 6H, $N(CH_3)_2$), 6.78 (d, *J*=8.3 Hz, 2H, Ar-H), 7.7 (d, *J* = 8.3 Hz, 2H, Ar-H), 7.77 (d, *J*=12.6 Hz, 1H), 7.93 (d, *J*=12.6 Hz ,1H), 8.13 (s, 2H, Benz-H), 13.66 (s, 1H, NH).		1349 (C-N), 1555 (C=N), 1641 (C=O), 3422 (N-H).
**5**	δ: 2.88 (s, 6H, $N(CH_3)_2$), 2.9 (s, 2H, pyrazole-H), 4.9 (s, 1H, NH), 6.72 (d, *J*=8.2 Hz, 2H, Ar-H), 7.2 (d, *J*=8.2 Hz, 2H, Ar-H), 7.6 (s, 1H, Benz-H), 7.88 (s, 1H, Benz-H)		
**6**	δ: 3.06 (s, 6H, $N(CH_3)_2$), 6.76 (s, 2H, NH_2), 6.87 (d, *J*=8 Hz, 2H, Ar-H), 7.61 (s, 1H, pyridine-H), 7.85 (d, *J*=8 Hz, 2H, Ar-H), 8.06 (s, 2H, Benz-H).		1610 (C=N), 2214 (C≡N), 3225 and 3356 (NH_2).
**7**	δ:2.9 (d, 1H, pyrane-H), 3.11 (s, 6H, $N(CH_3)_2$), 4.5 (s, 2H, NH_2), 6.86 (d, *J*=8.6 Hz, 2H, Ar-H), 7.2 (d, *J*=8 Hz, 1H, pyrane-H), 7.84 (d, *J*=8.6 Hz, 2H, Ar-H), 8.06 (s, 2H, Benz-H).	δ: 44.59 (CH_3), 68.87 (CH), 112.20 (CH), 116.88 (CH_2), 119.04 (CH), 133.78 (CH_2), 133.78 (CH_2), 154.82 (CH_2), 159.32 (CH).	1611 (C=N), 2208 (C≡N), 3225 and 3424 (NH_2).
**8**	δ: 2.81 (s, 6H, $N(CH_3)_2$), 3.0 (d, *J*=6.5 Hz, 2H, isoxazoline-H), 5.75 (t, 1H, isoxazoline-H), 7.11 (d, *J*=8.2 Hz, 2H, Ar-H), 7.27 (d, *J*=8.2 Hz, 2H, Ar-H), 7.72 (s, 1H, Benz-H), 8.03 (s, 1H, Benz-H).		

**9**	δ: 2.68 (s, 3H, $COCH_3$), 6.19 (s, 2H, $COCH_2$), 7.63 (d, *J*=8.2 Hz, 2H, Ar-H), 7.86 (s, 1H, Benz-H), 8.0 (s, 1H, Benz-H), 8.27 (d, *J*=8.2 Hz, 2H, Ar-H).		1654, 1689 (C=O).
**10**	δ:2.02 (s, 3H, CH_3), 7.09 (d, *J*=8 Hz, 2H, Ar-H), 7.2 (d, *J*=8 Hz, 2H, Ar-H), 7.4 (d, *J*=8 Hz, 2H, Ar-H), 7.46 (d, *J*=8 Hz, 2H, Ar-H), 8.95 (s, 2H, methelene-H), 8.69 (s, 1H, Benz-H), 8.86 (s, 1H, Benz-H), 9.7 (s, 1H, pyrazanobenzimidazole-H).	δ:20.9, 102.2, 112.4, 117.1, 119.5, 124.2, 128.9, 129.9, 130.5, 131.1, 131.5, 132.1, 134.2, 138.5, 145.1, 167.3.	
**11**	δ: 2.06 (s, 3H, CH_3), 7.18 – 7.41 (m, 4H, Ar-H), 7.58 (d, *J*= 7.4 Hz, 2H, Ar-H), 7.68 (d, *J*=7.6 Hz, 2H, Ar-H), 8.13 (s, 1H, Benz-H), 8.25 (s, 1H, Benz-H), 8.85 (s, 2H, methelene-H), 8.91 (s, 1H, pyrazanobenzimidazole-H).		
**12**	δ:4.64 (s, 2H, CH_2-Ph), 7.26-7.48 (m, 5H, Ar-H), 7.67 (d, *J*=7.2 Hz, 2H, Ar-H), 7.8 (d, *J*=7.2 Hz ,2H, Ar-H), 8.07 (s, 2H , Benz-H), 8.52 (s, 2H, methelene-H), 8.77 (s, 1H, byrazanobenzimidazole-H)	δ: 64.73 (CH_2), 101.23 (CH), 112.02 (CH), 115.08 (CH), 120.65 (CH), 123.35 (CH_2), 124.97 (CH_2), 127.31 (CH), 128.93 (CH), 131.08 (CH), 132.34 (CH), 136.66 (CH_2), 139.54 (CH_2), 140.25 (CH), 142.96 (CH_2), 144.21 (CH_2), 162.19 (CH).	

**13**	δ: 1.25 (s, 3H, CH_3), 7.77 (d, *J*=7.7 Hz, 2H, Ar-H), 8.1 (d, *J*=7.7 Hz, 2H, Ar-H), 8.33 (s, 1H, Benz-H), 8.93(s, 1H, Benz-H), 9.68 (s, 1H, pyrazanobenzimidazole-H).		
**14**	δ: 7.19 (d, *J*=7.7 Hz, 2H, Ar-H), 7.3 (d, *J*=7.7 Hz, 2H, Ar-H), 7.74 (s, 2H, Benz-H), 8.11 (s, 1H, pyridinonobenzimidazole-H), 8.85 (s, 1H, pyridinobenzimidazole-H), 9.07 (s, 1H, OH).		1219-1296 (C-O),1557-1642 (C=C,C=N), 3448 (OH).

^{1}H NMR of compound 4

1H NMR

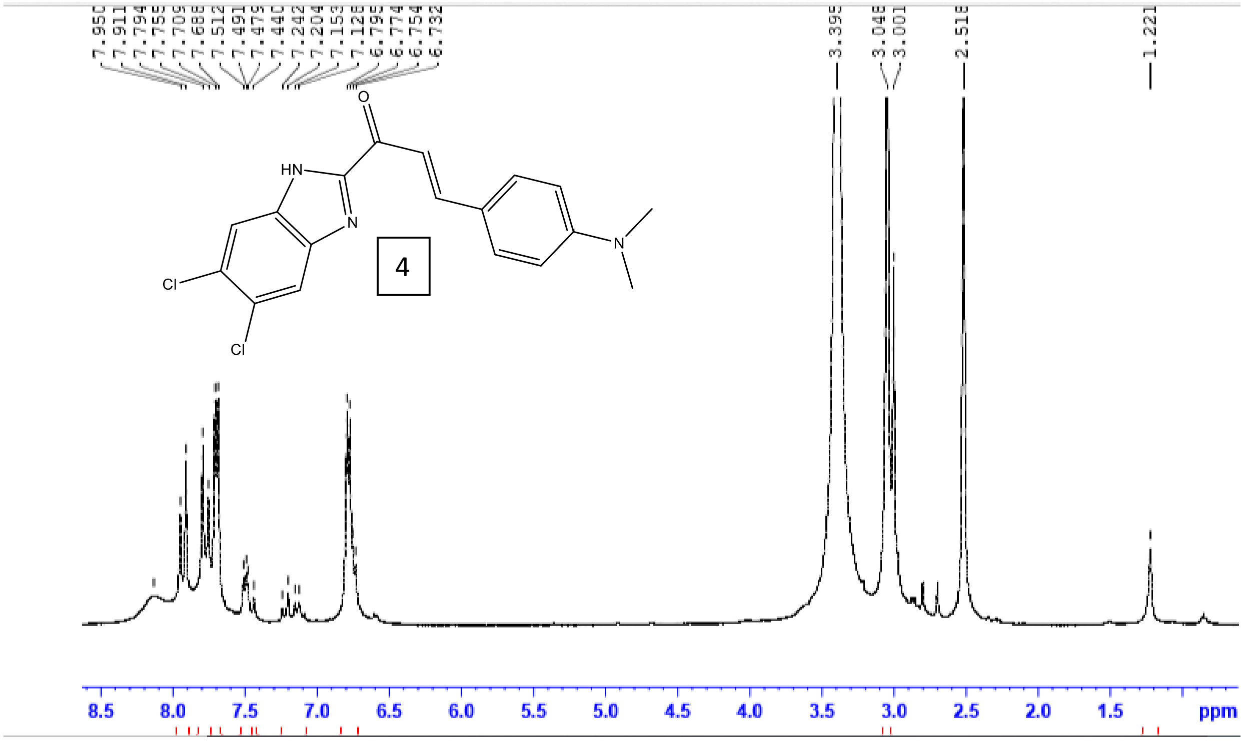

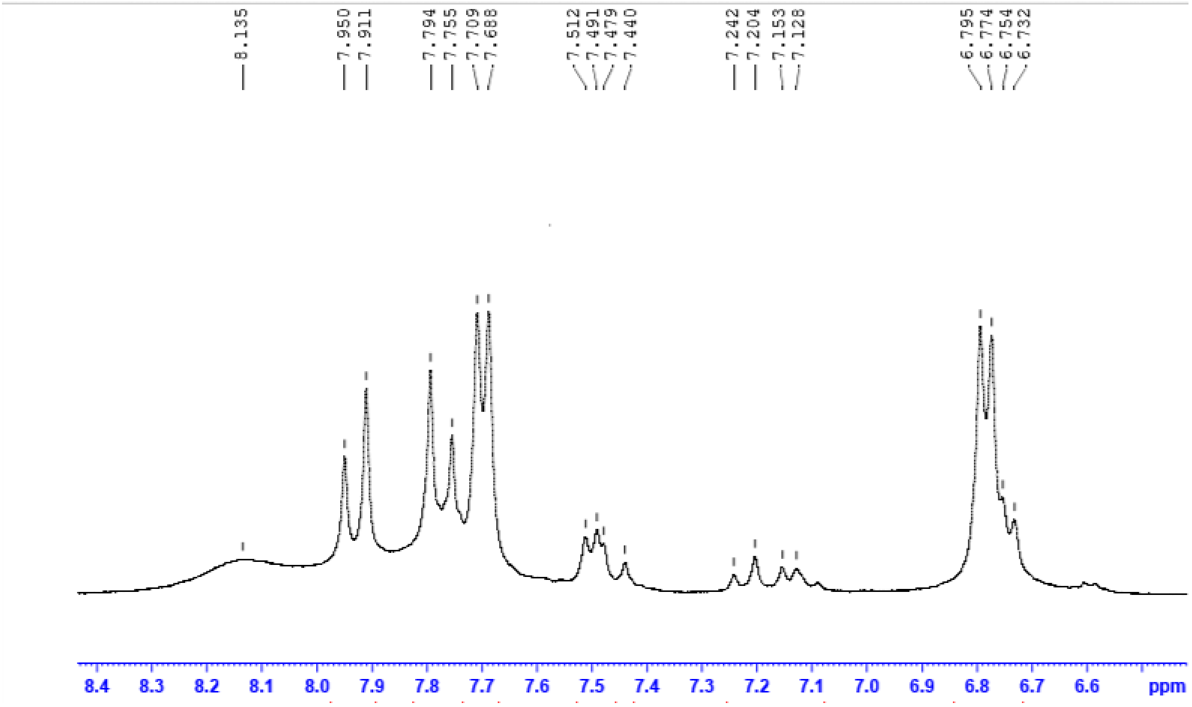

IR of compound 4

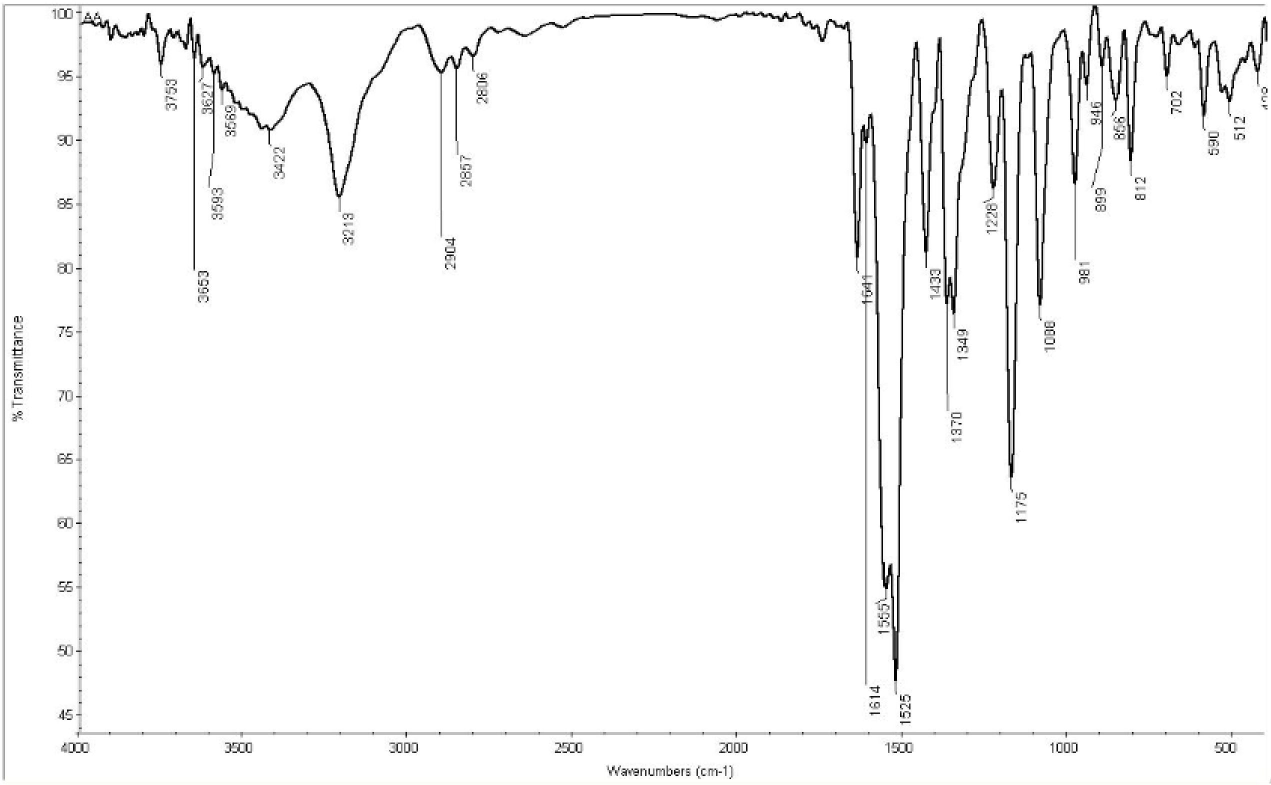

Mass of compound 4

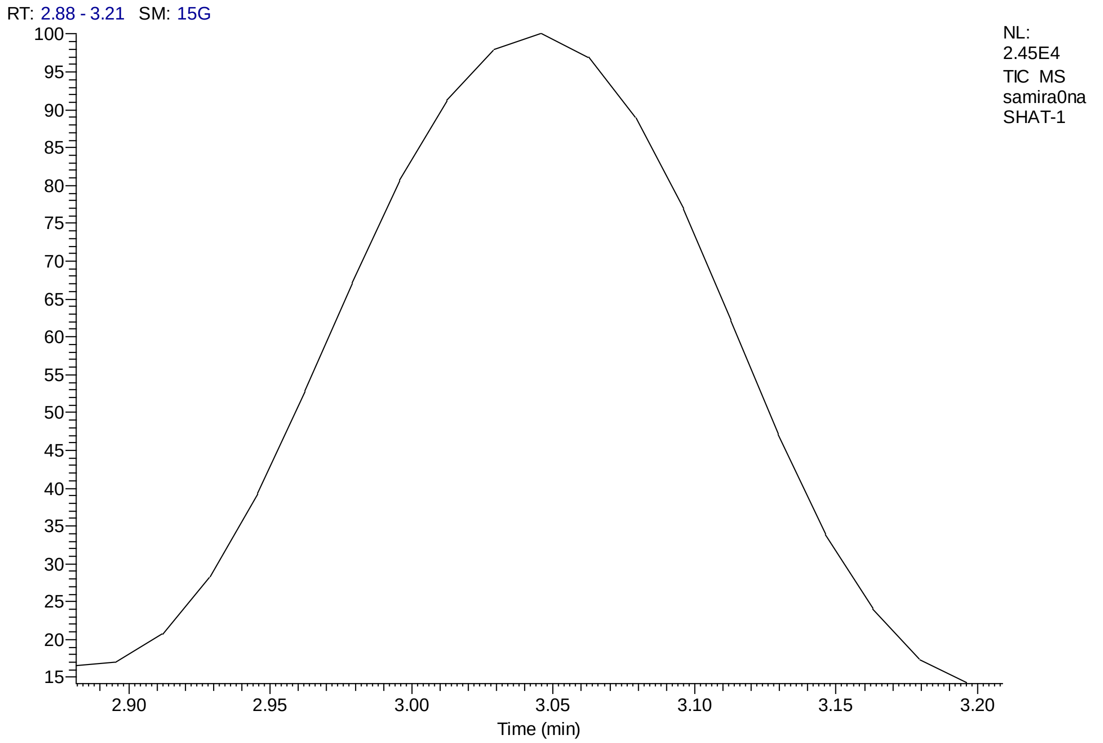

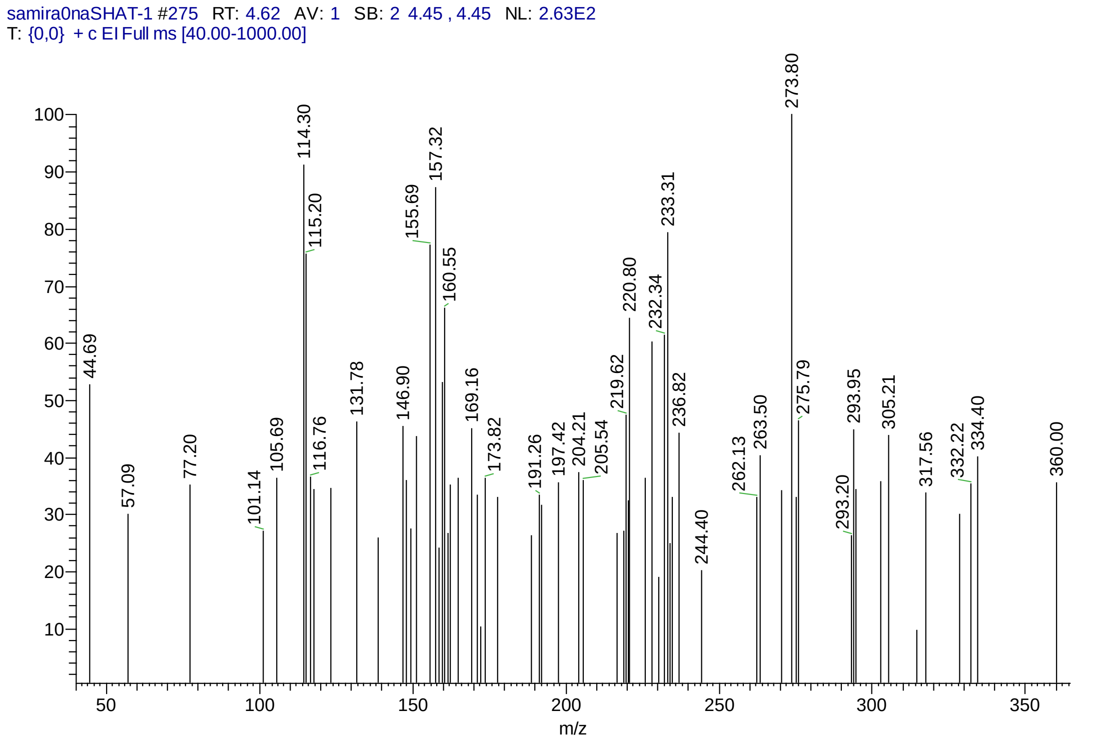

^{1}H NMR of compound 5

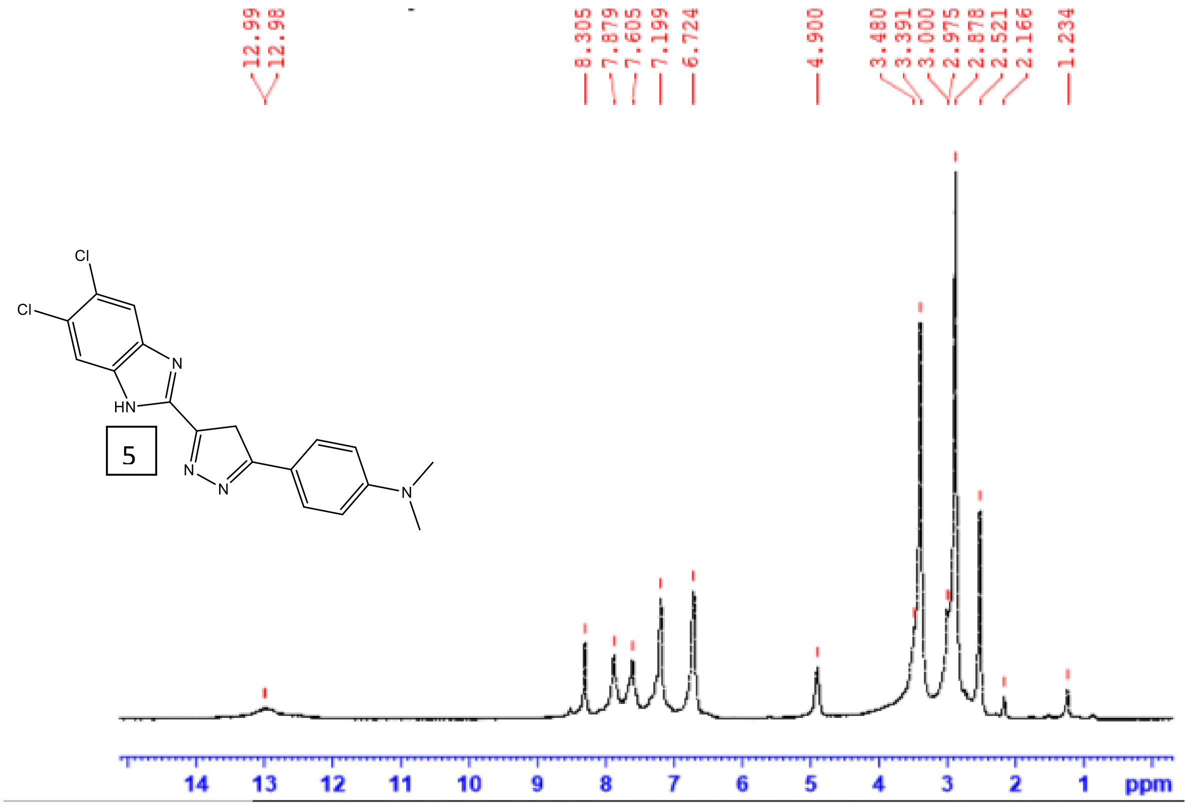

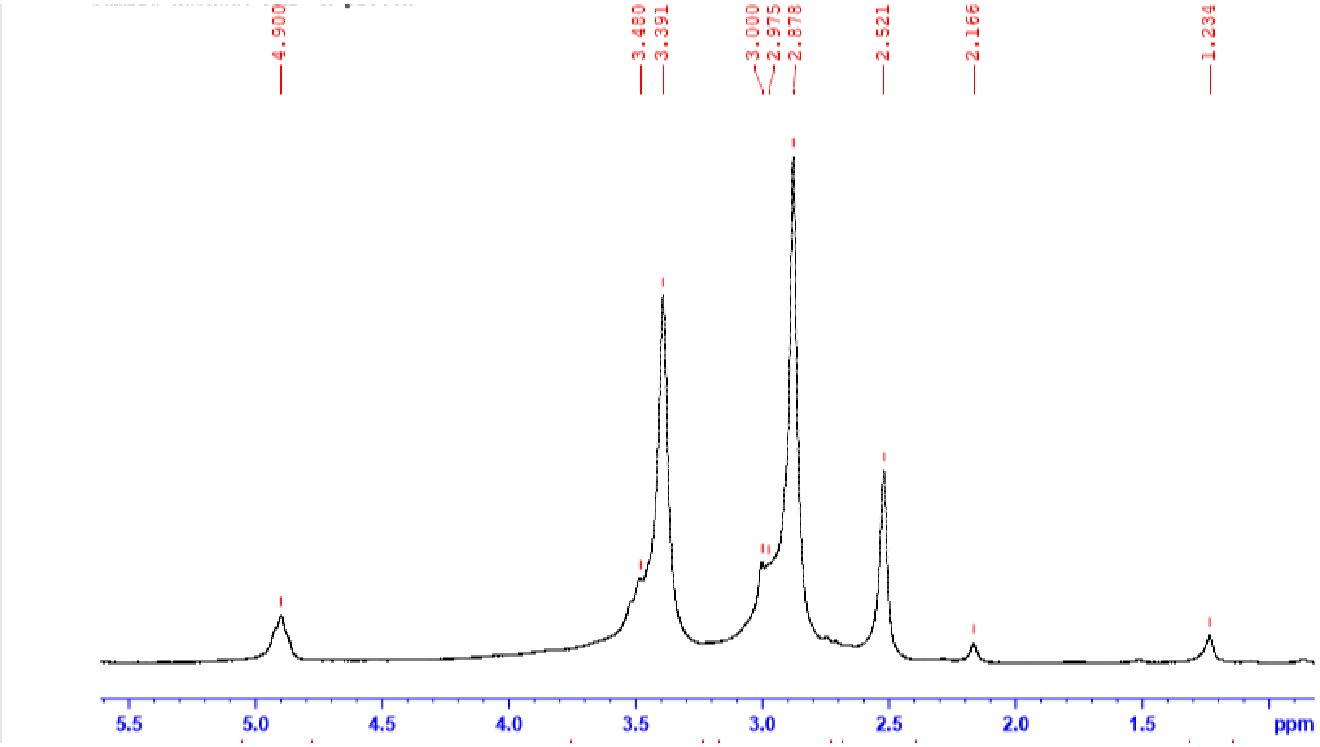

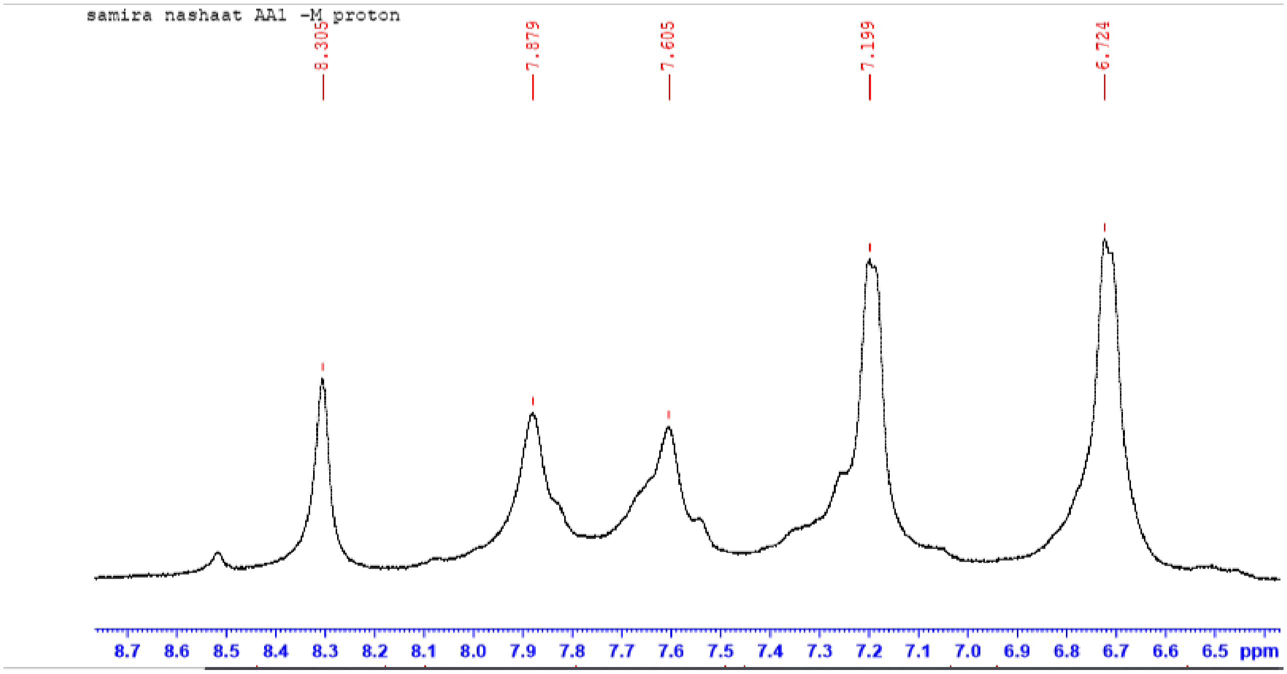

^{13}C NMR of compound 5

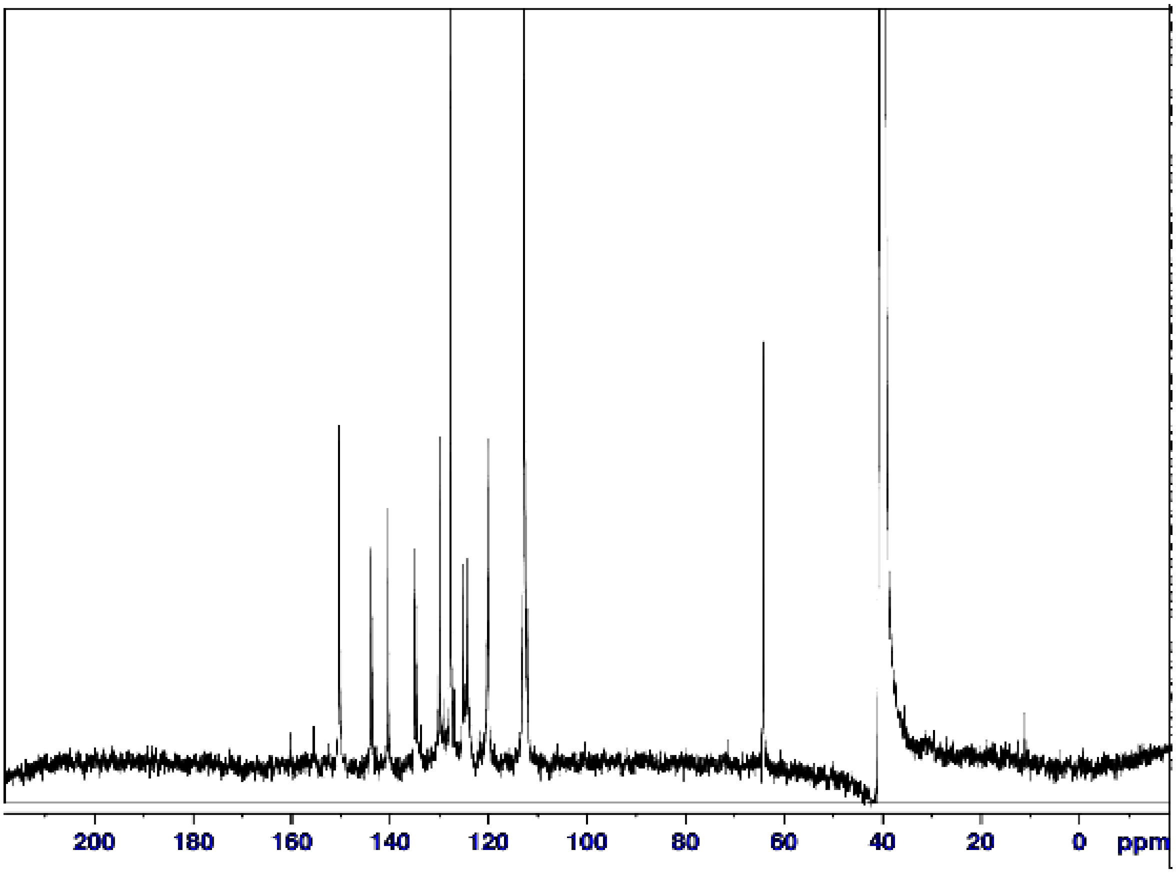

APT of compound 5

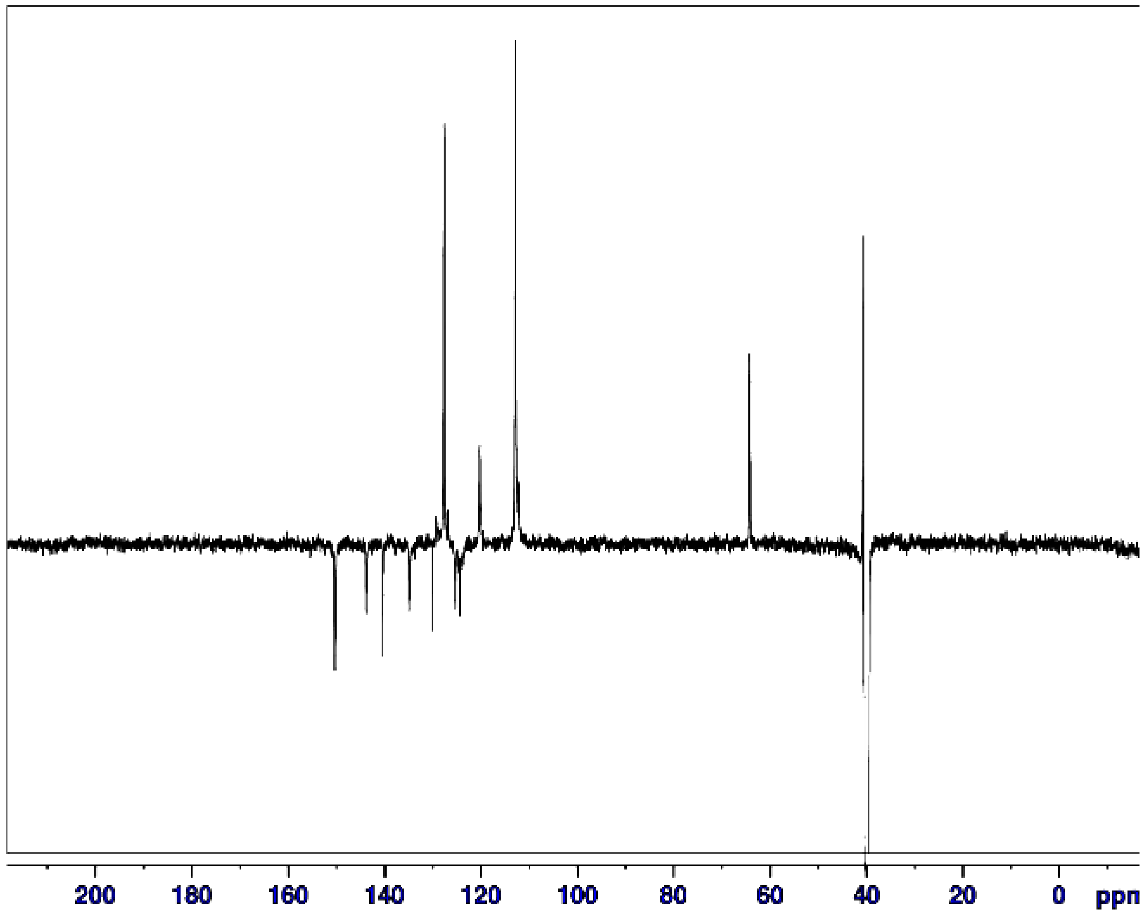

Mass of compound 5

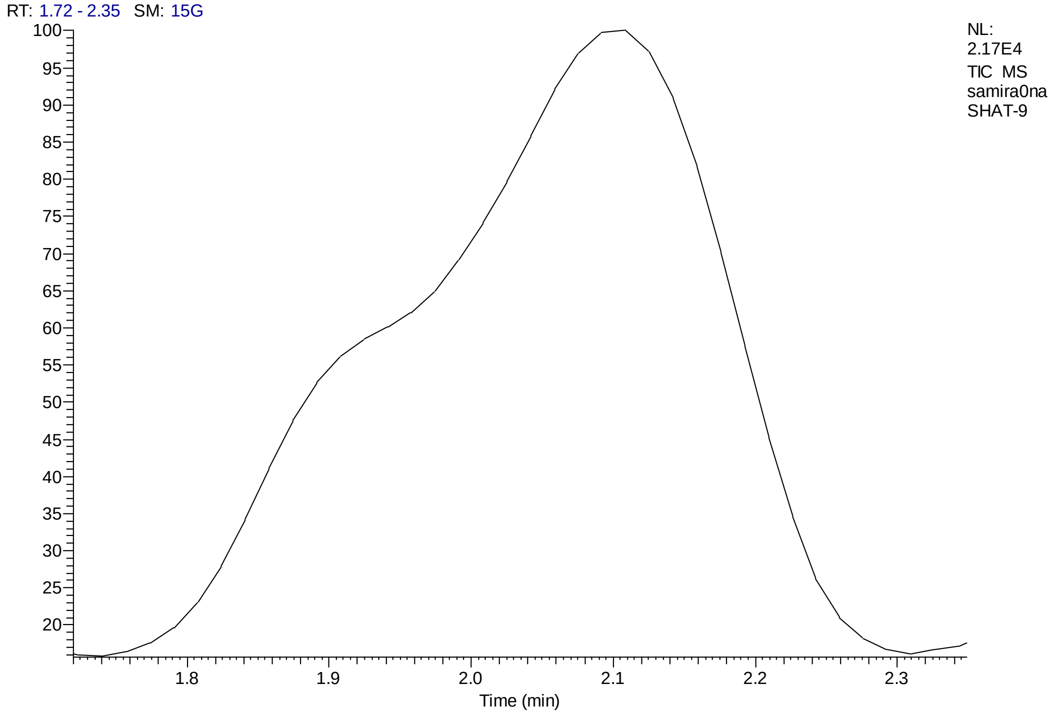

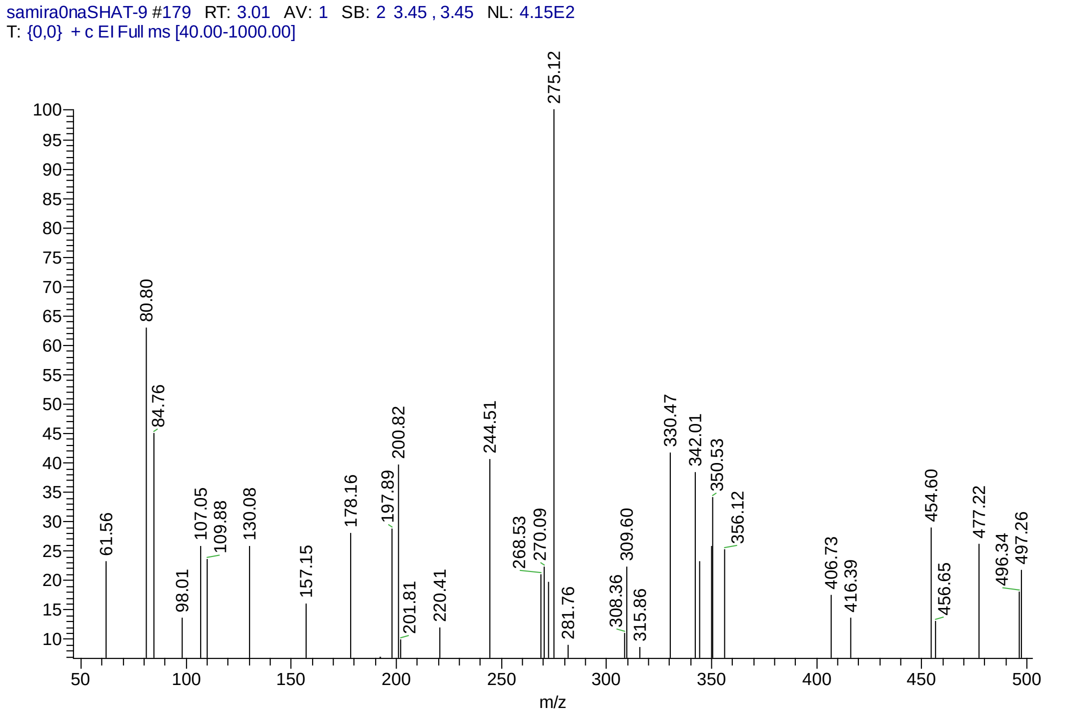

^{1}H NMR of compound 6

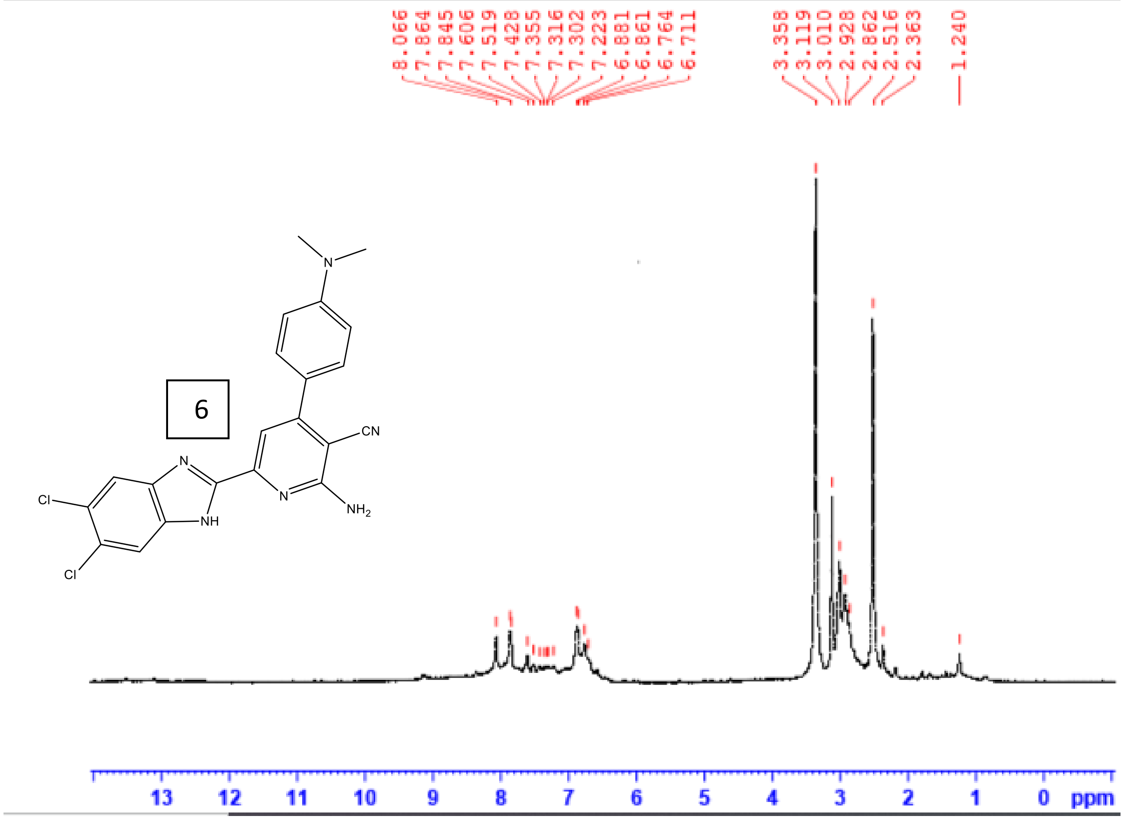

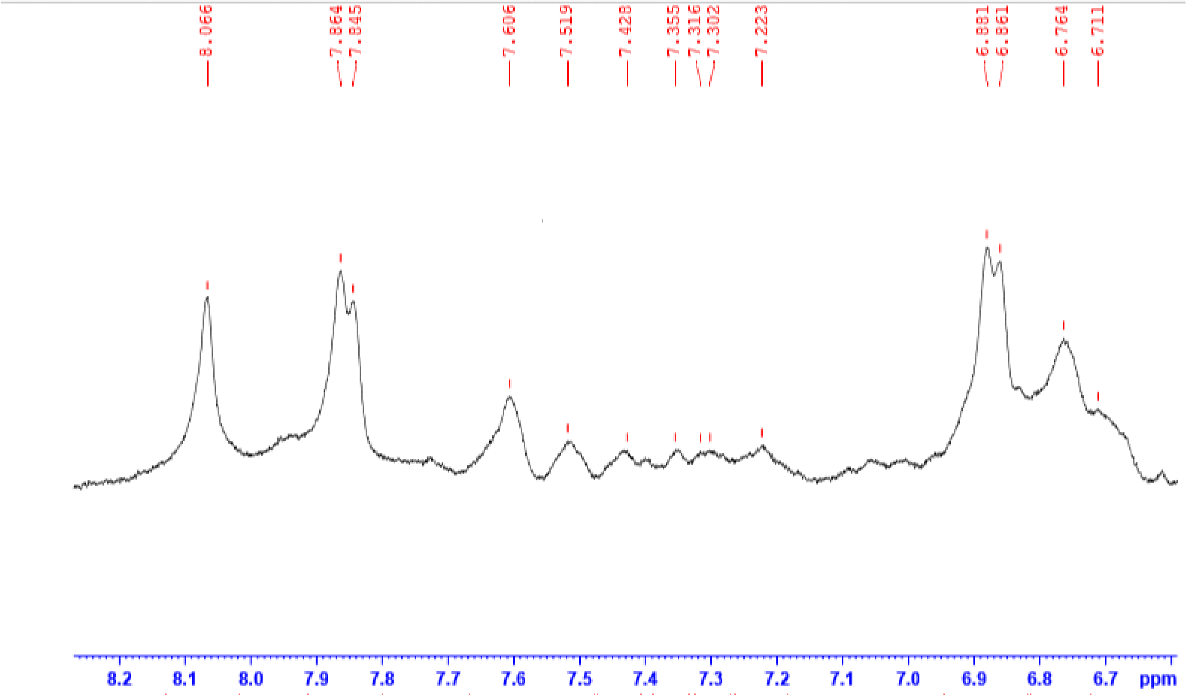

IR of compound 6

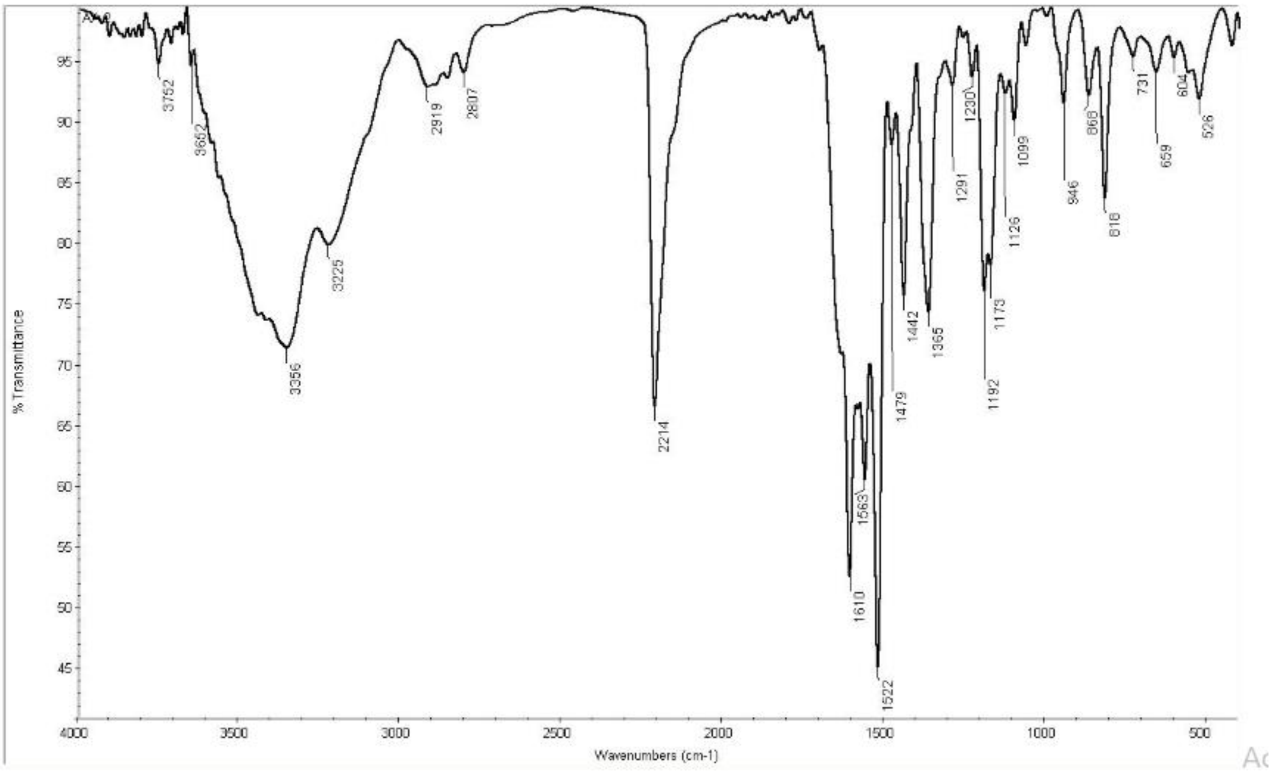

Mass of compound 6

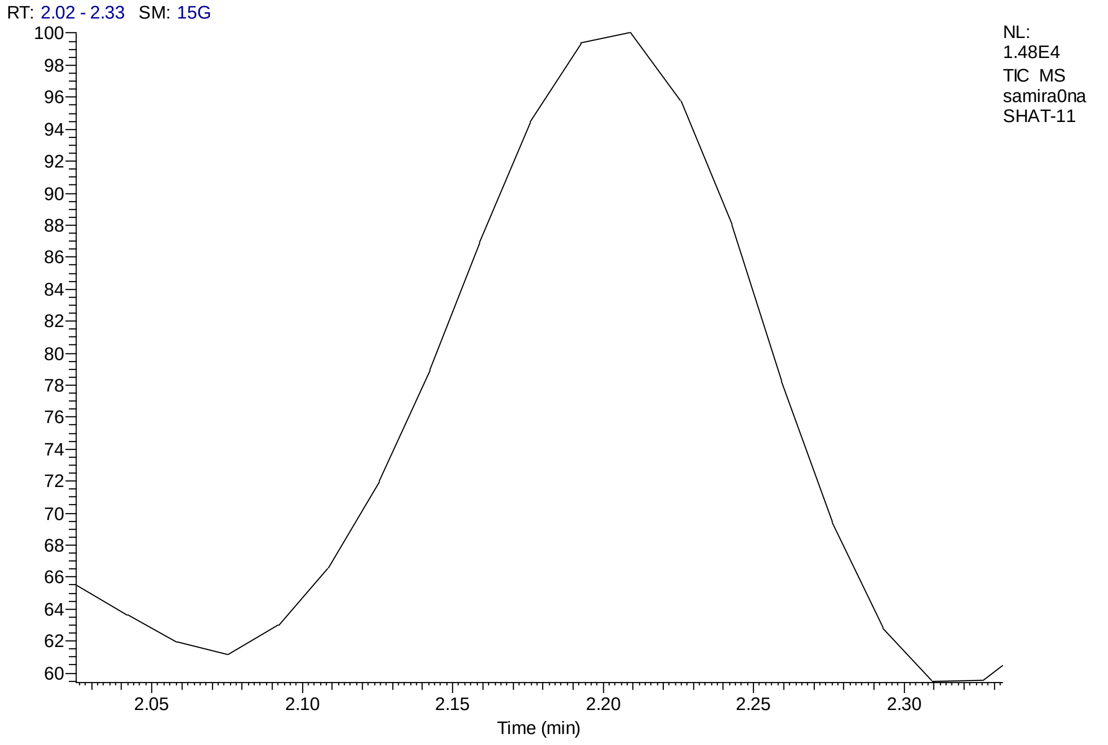

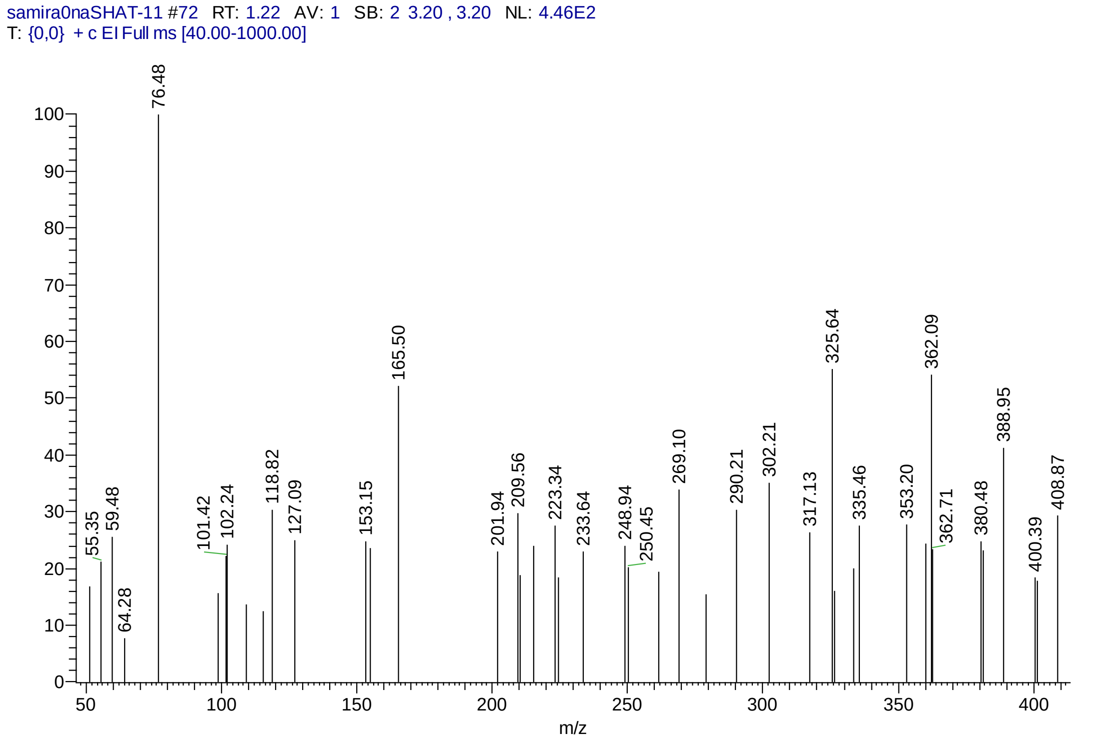

^{1}H NMR of compound 7

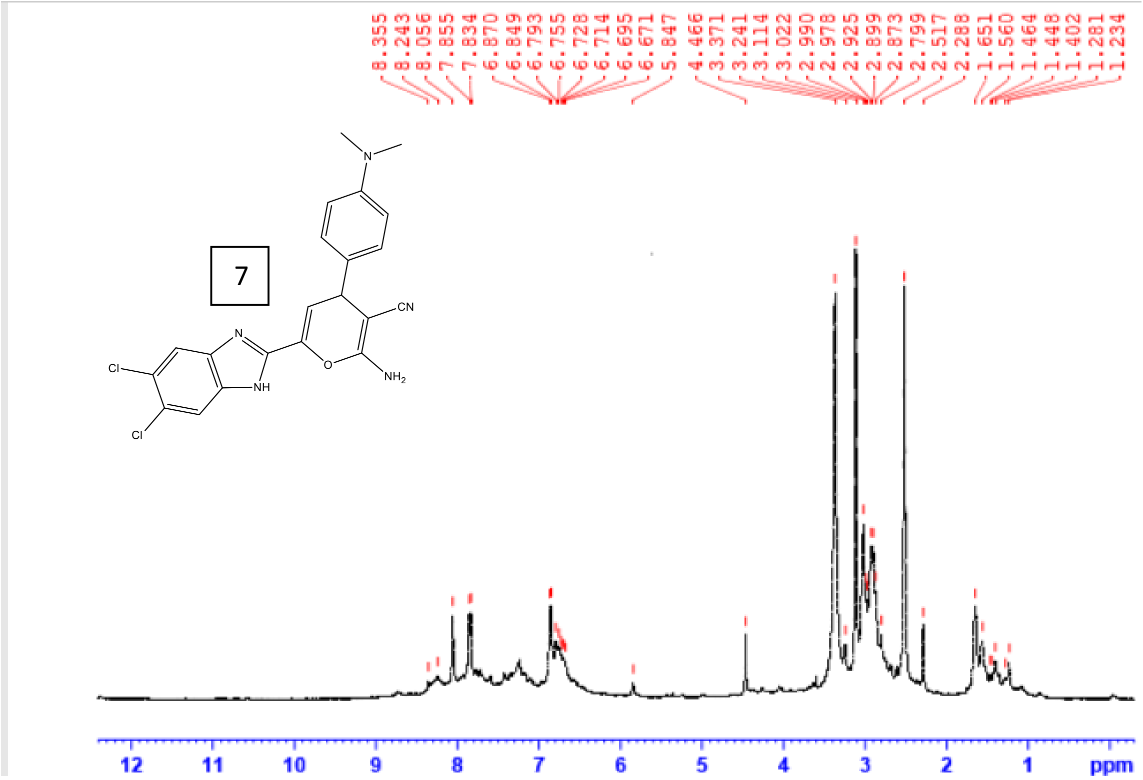

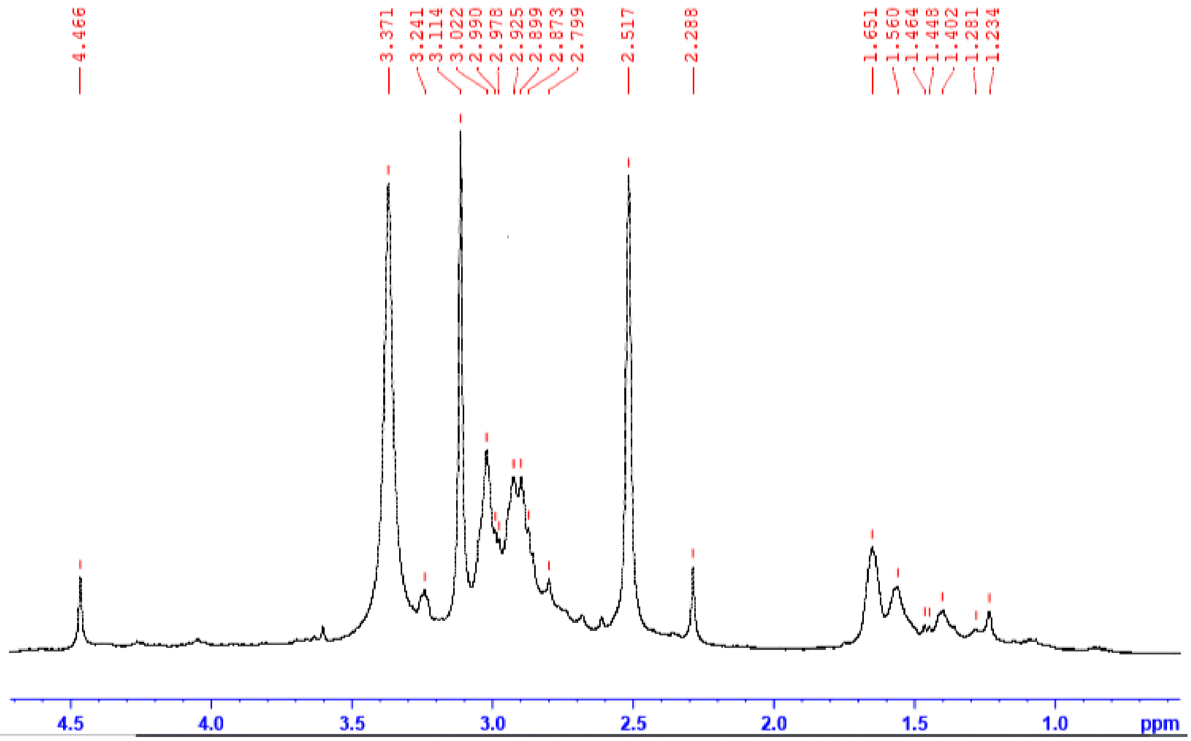

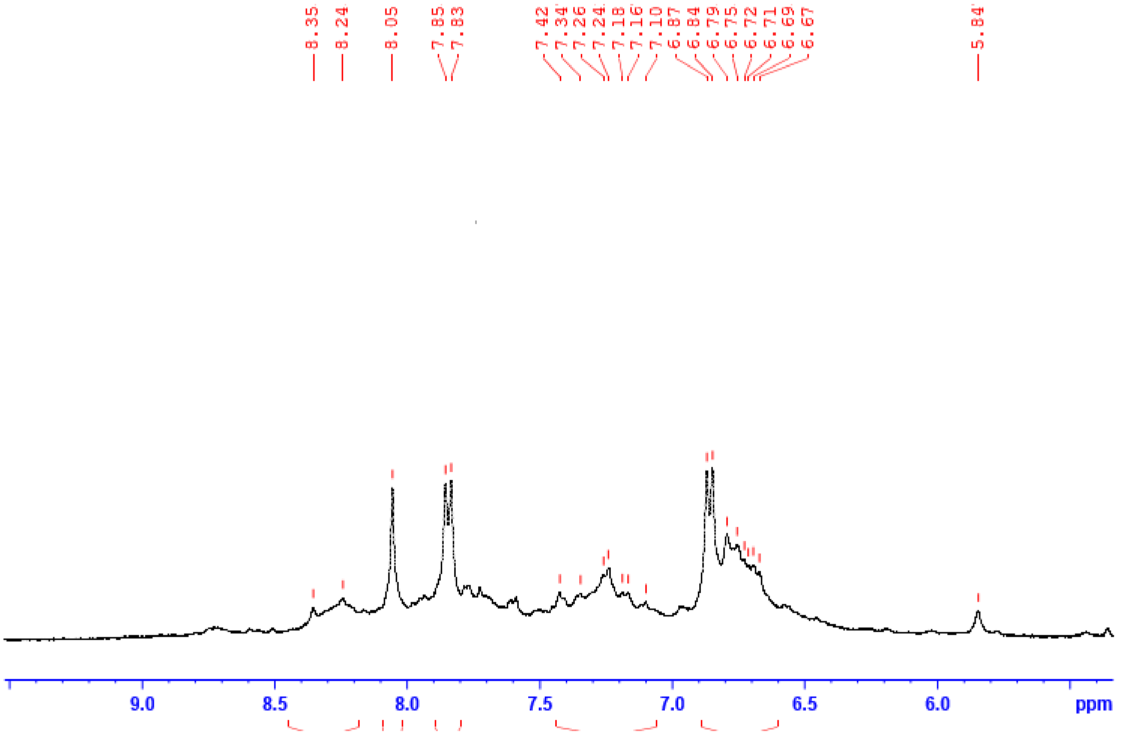

IR of compound 7

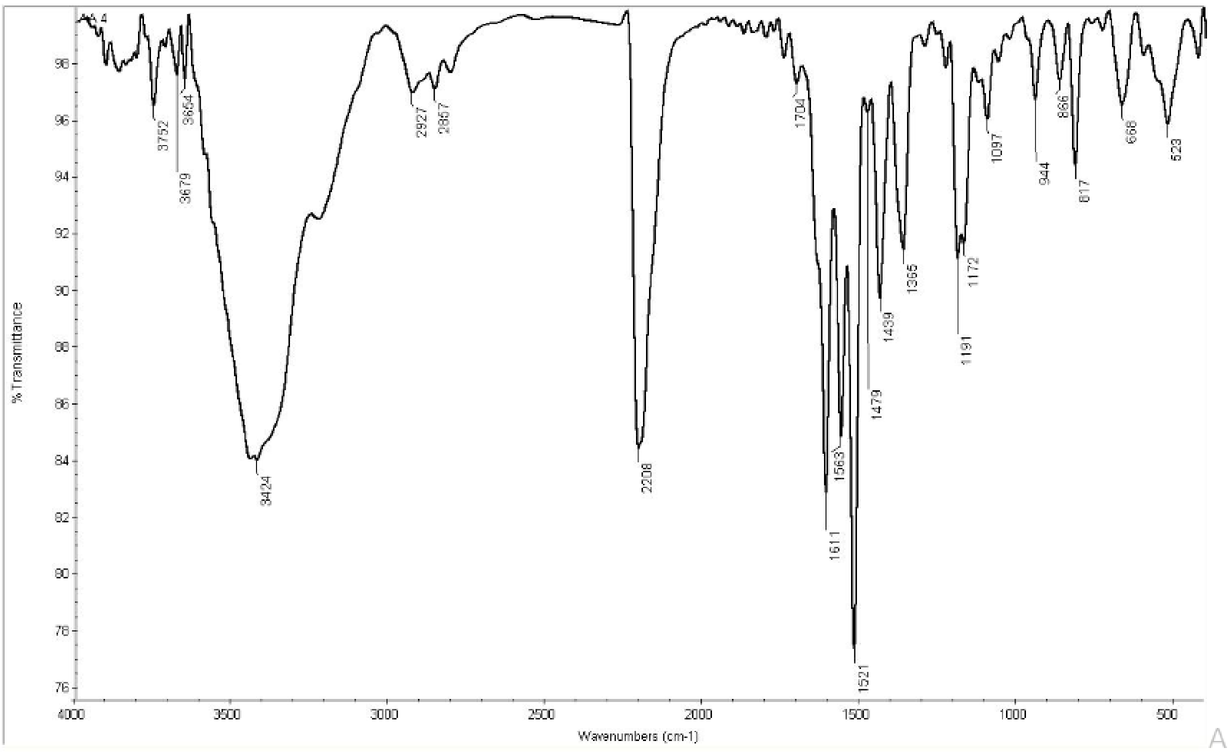

^{13}C NMR of compound 7

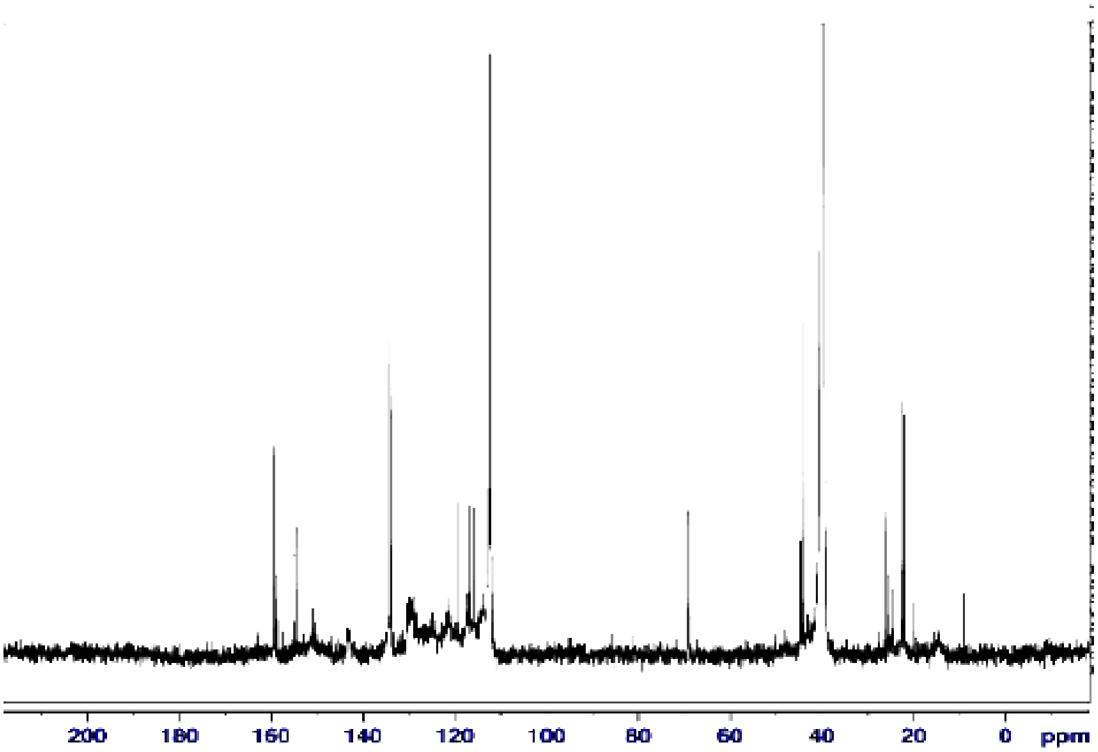

APT of compond 7

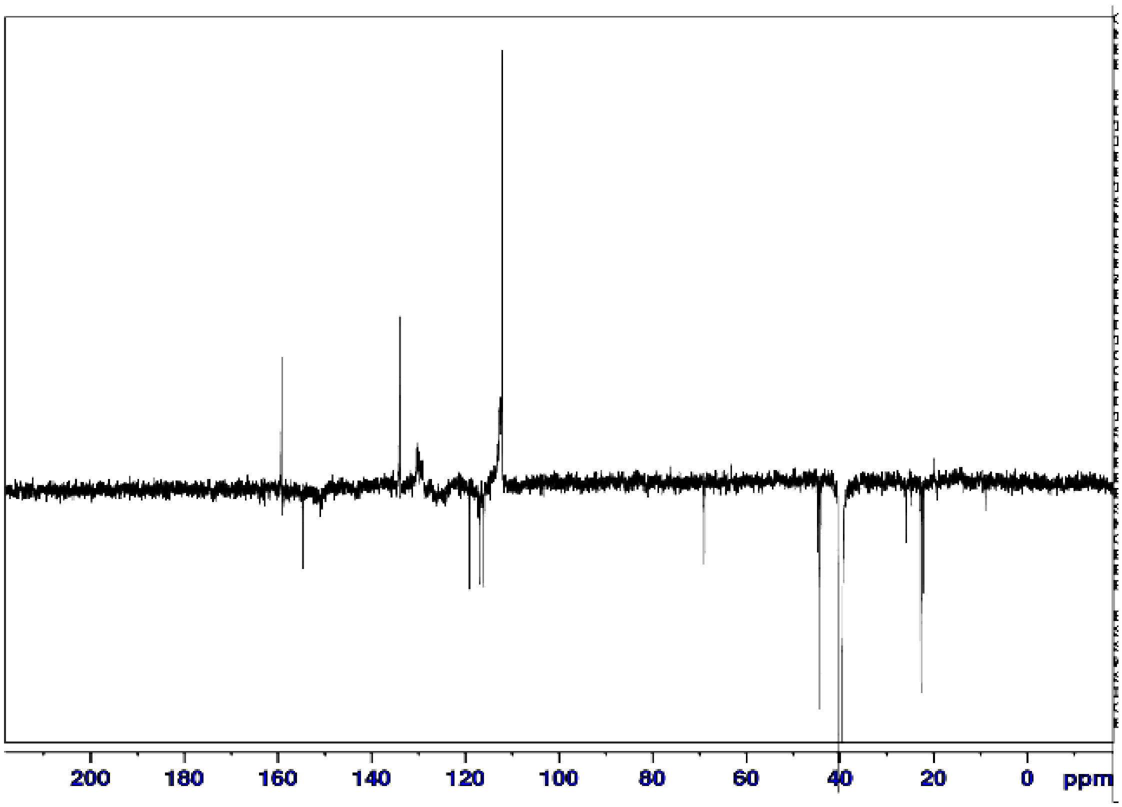

Mass of compound 7

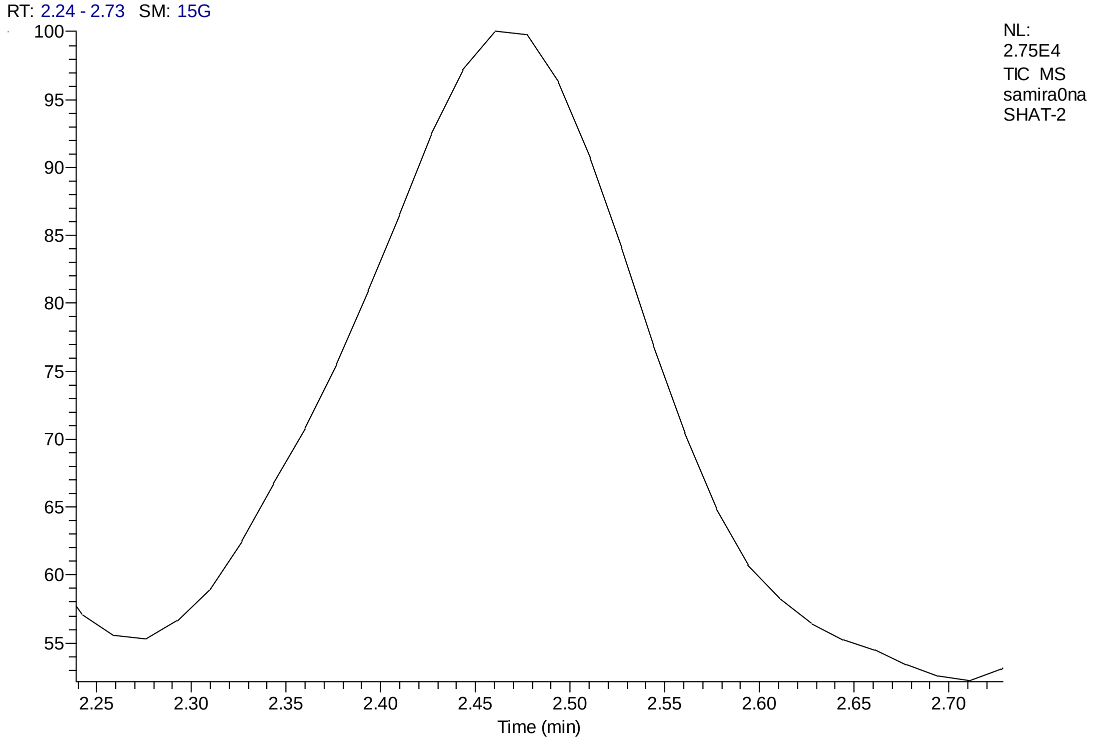

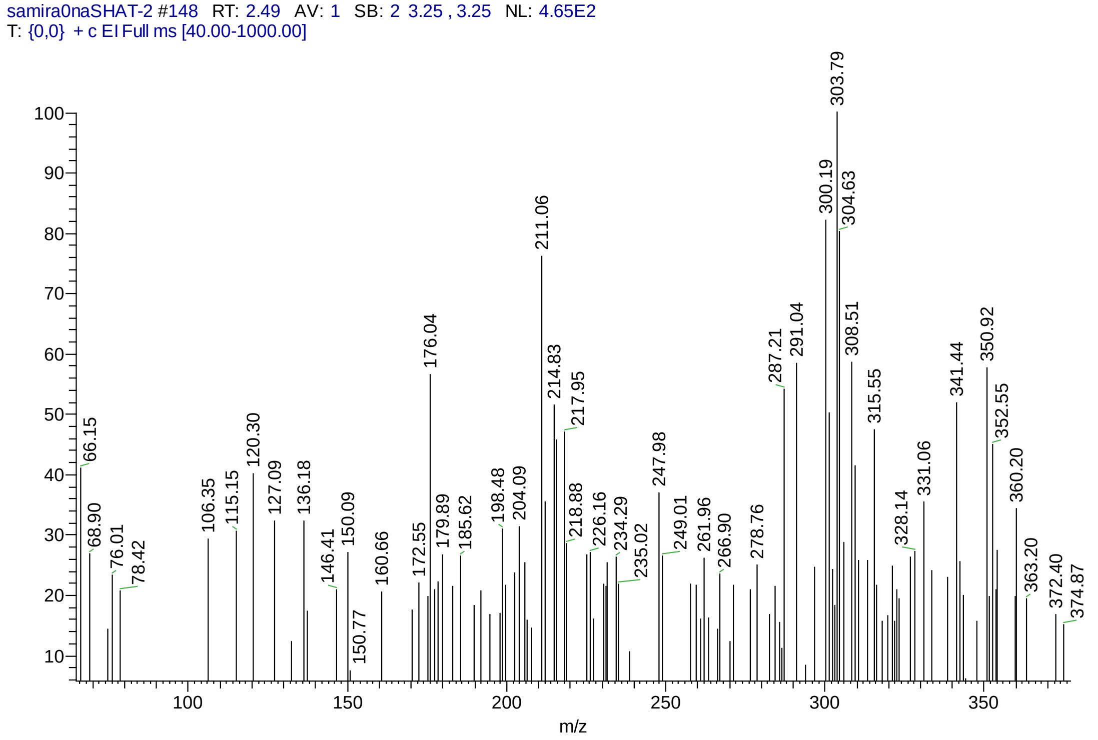

¹H NMR of compound 8

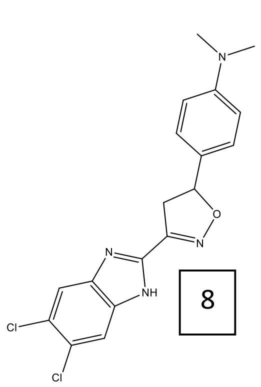

8

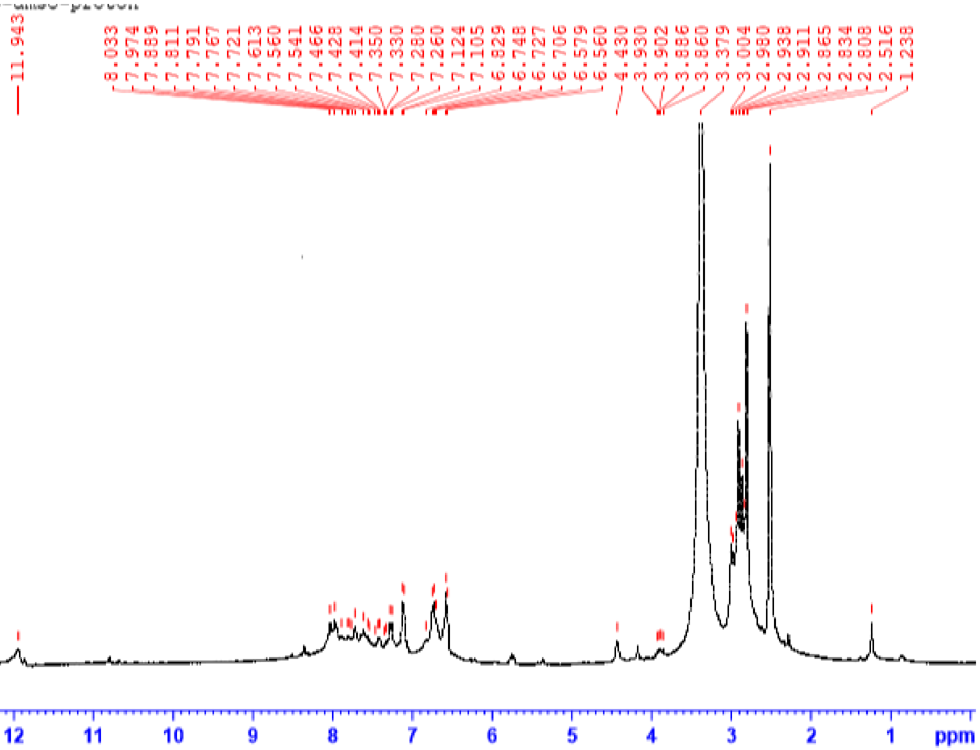

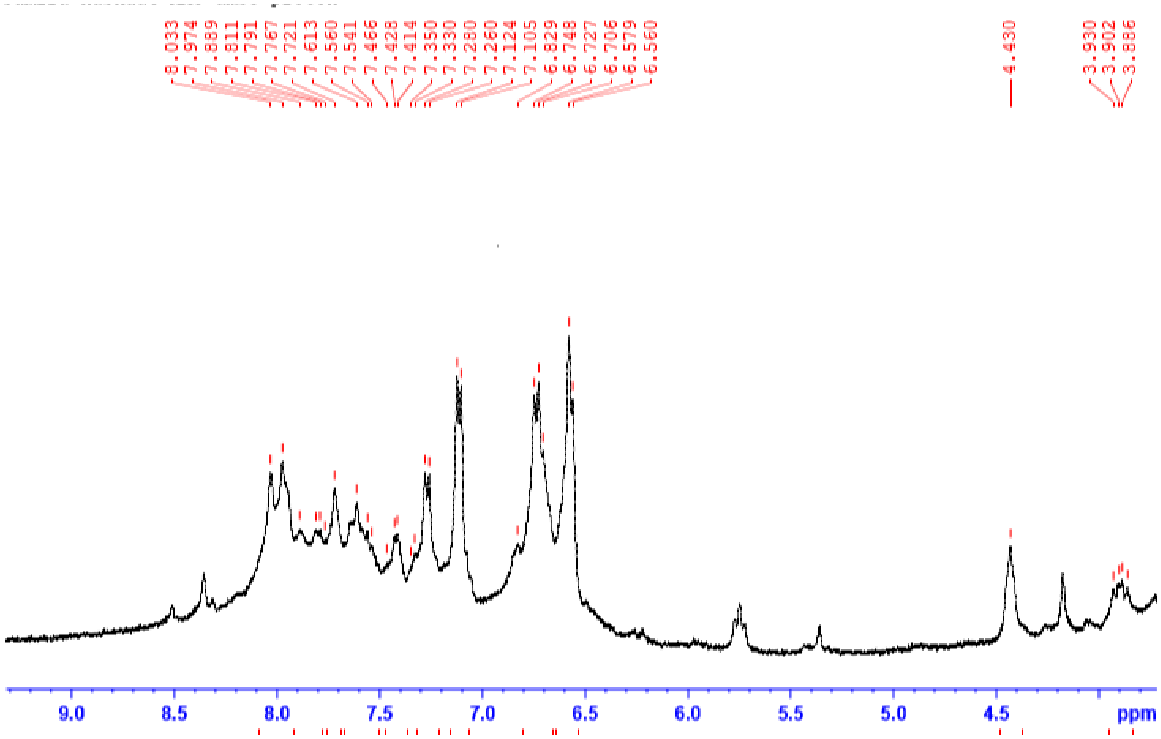

^{13}C NMR of compound 8

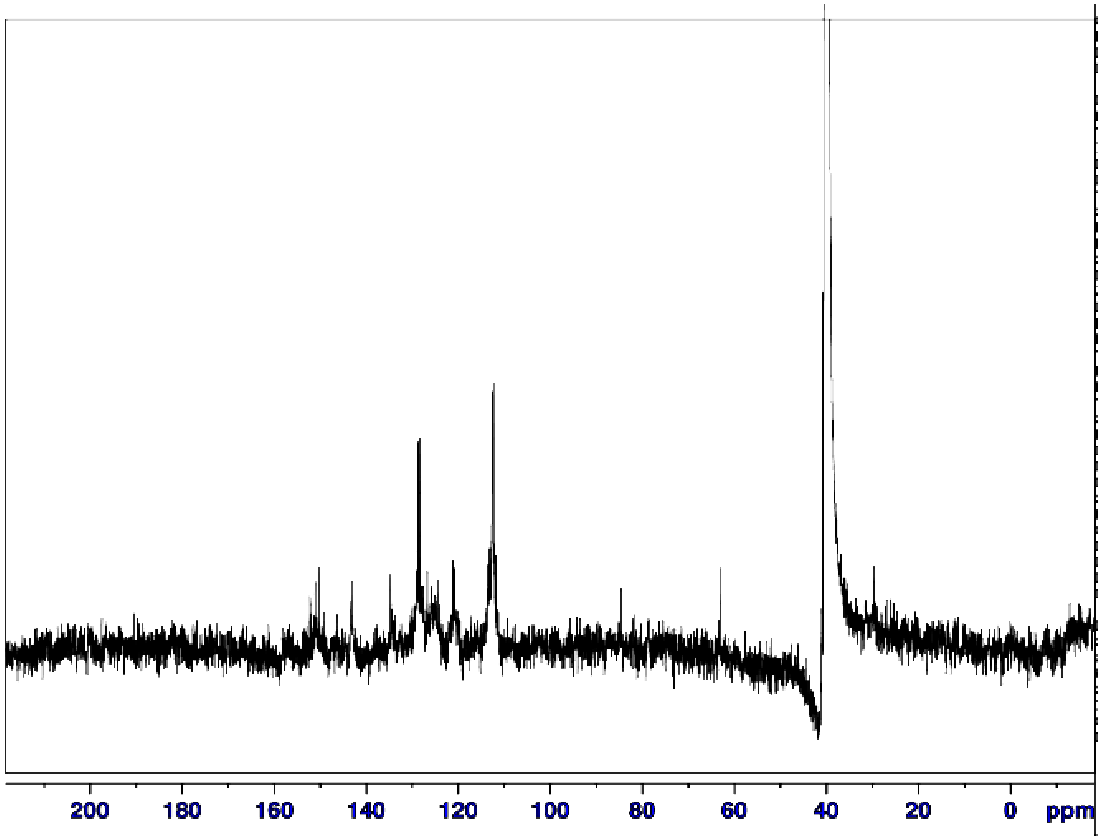

APT of compound 8

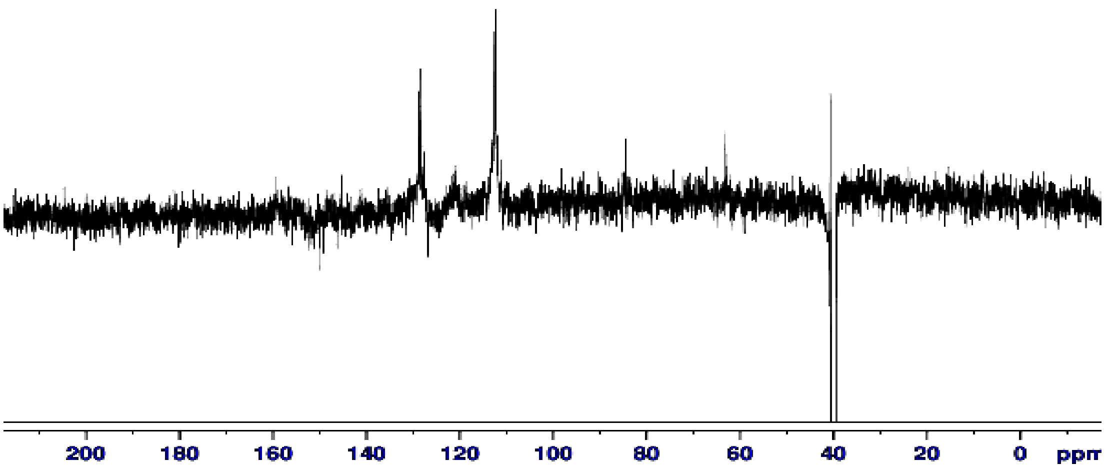

Cosy of compound 8

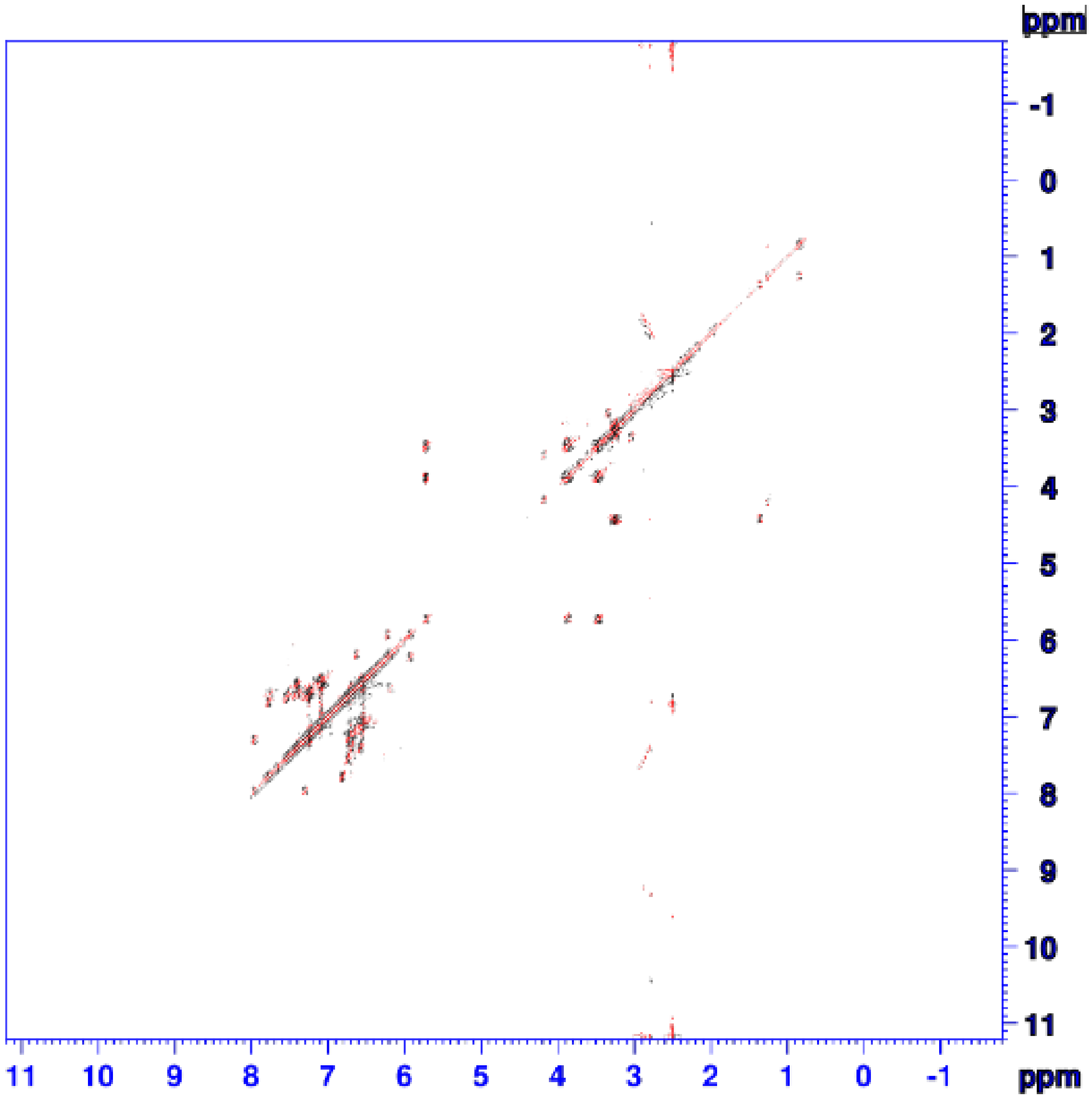

Mass of compound 8

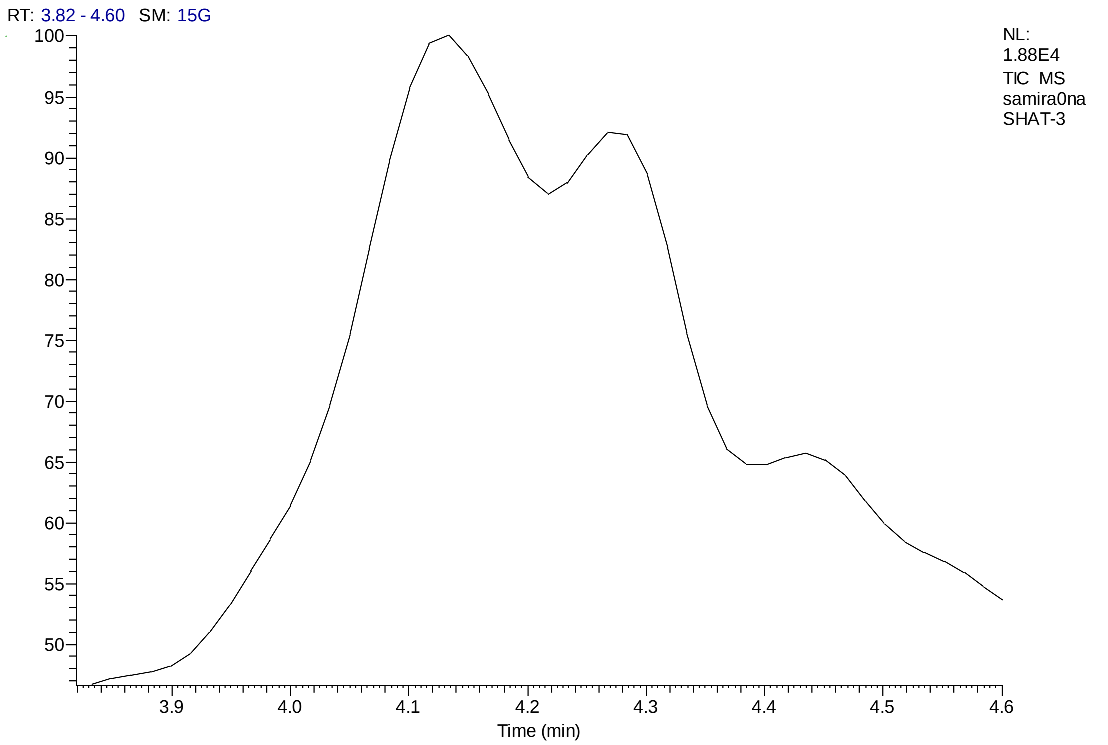

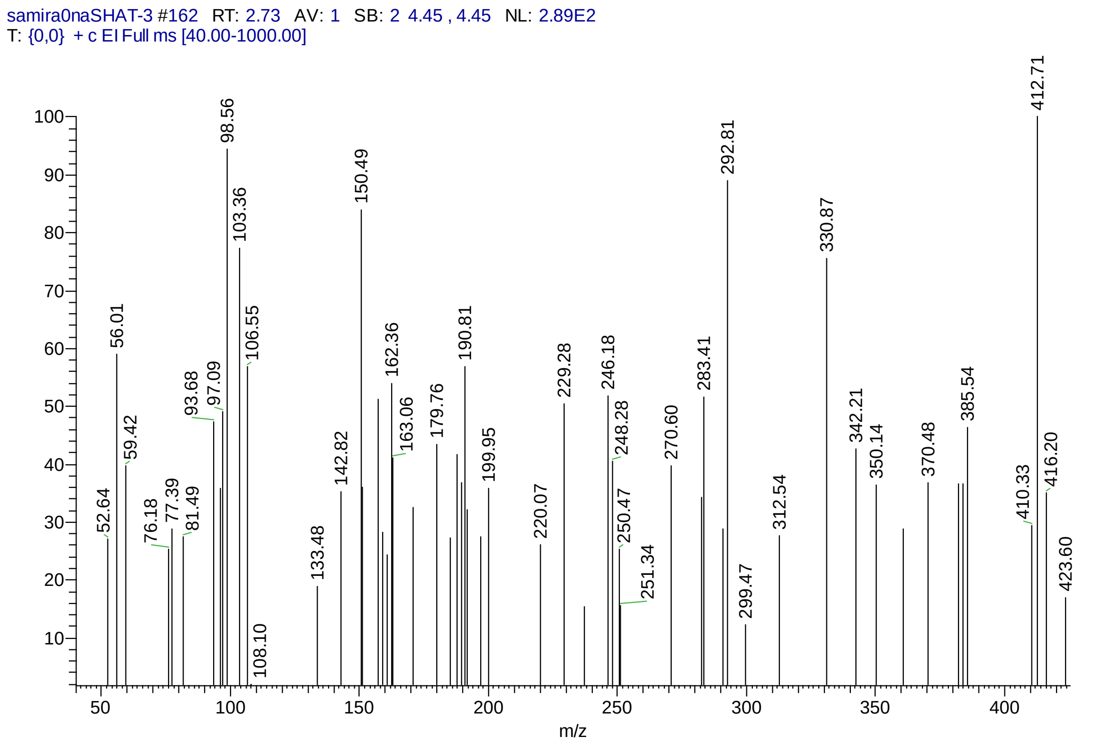

^{1}H NMR of compound 9

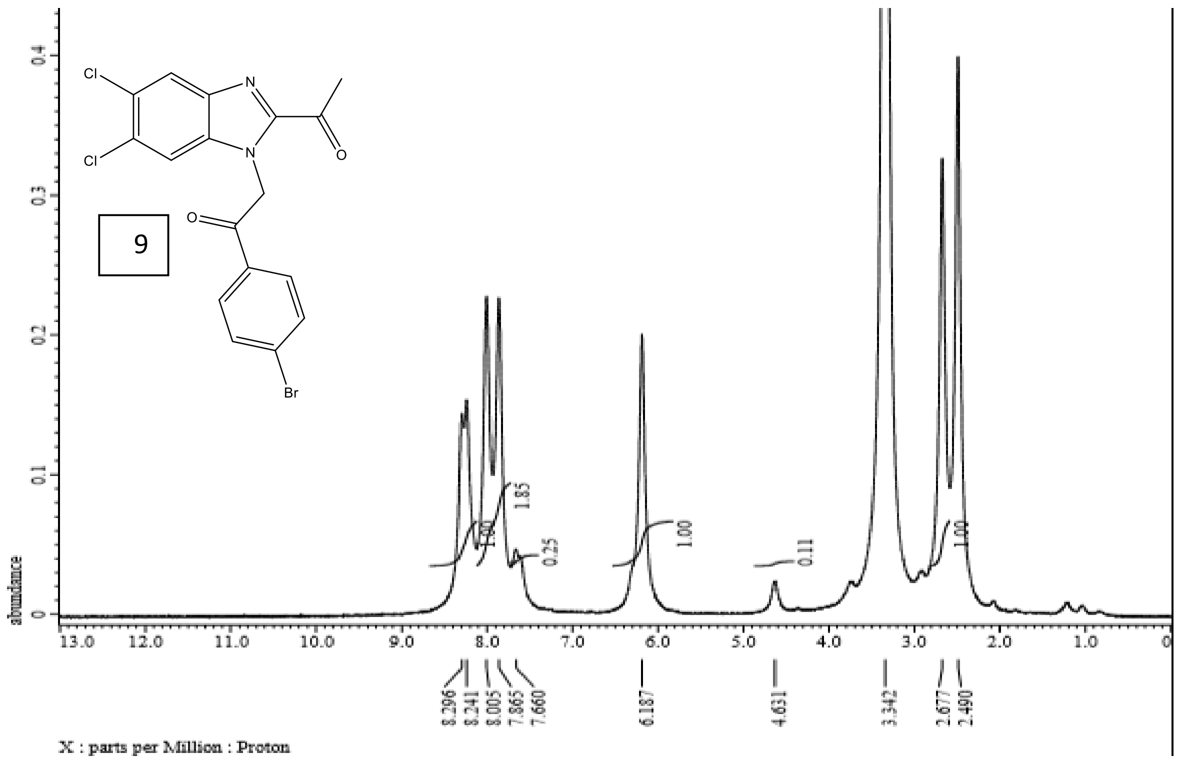
9
Cl
Cl
N
N
O
O
Br
abundance
X : parts per Million : Proton
8.296
8.241
8.005
7.865
7.660
6.187
4.631
3.342
2.677
2.490
1.00
1.85
0.25
1.00
0.11
1.00

IR of compound 9

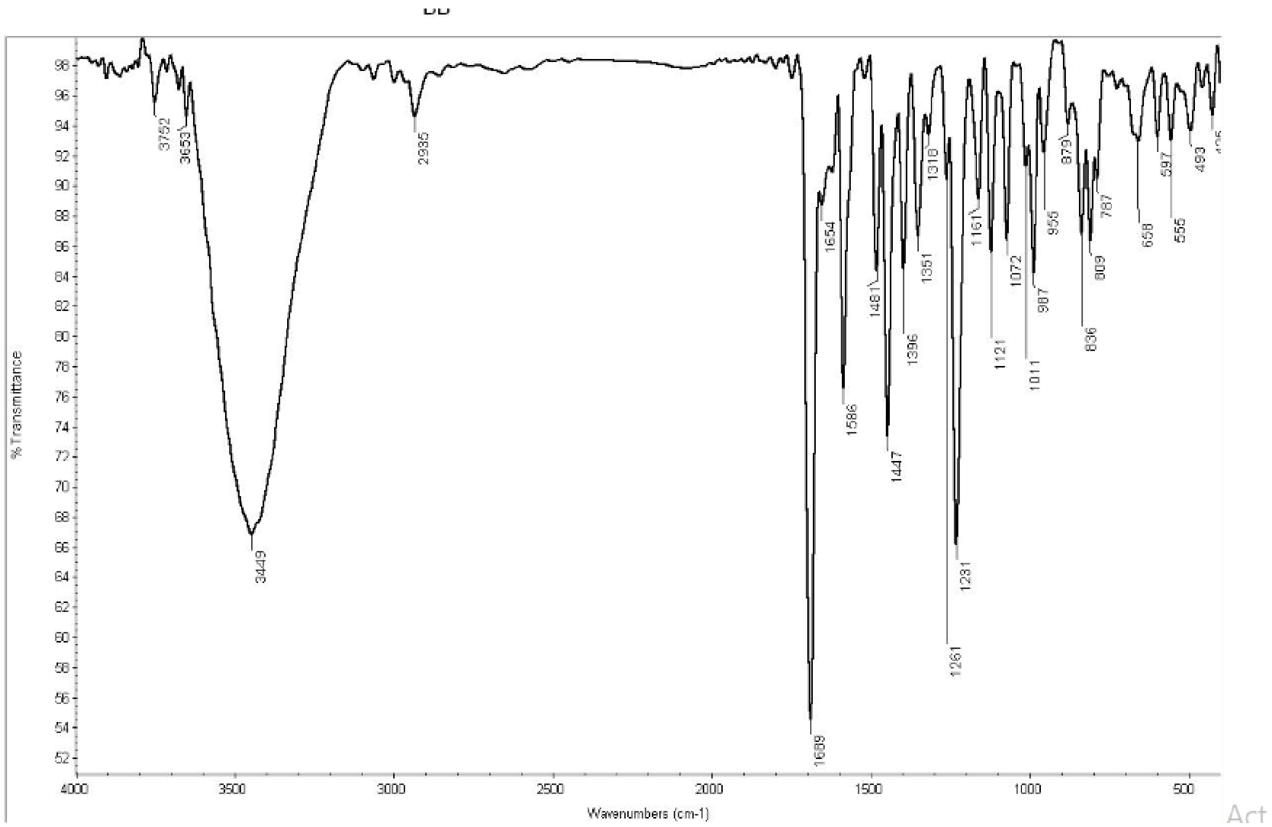

Mass of compound 9

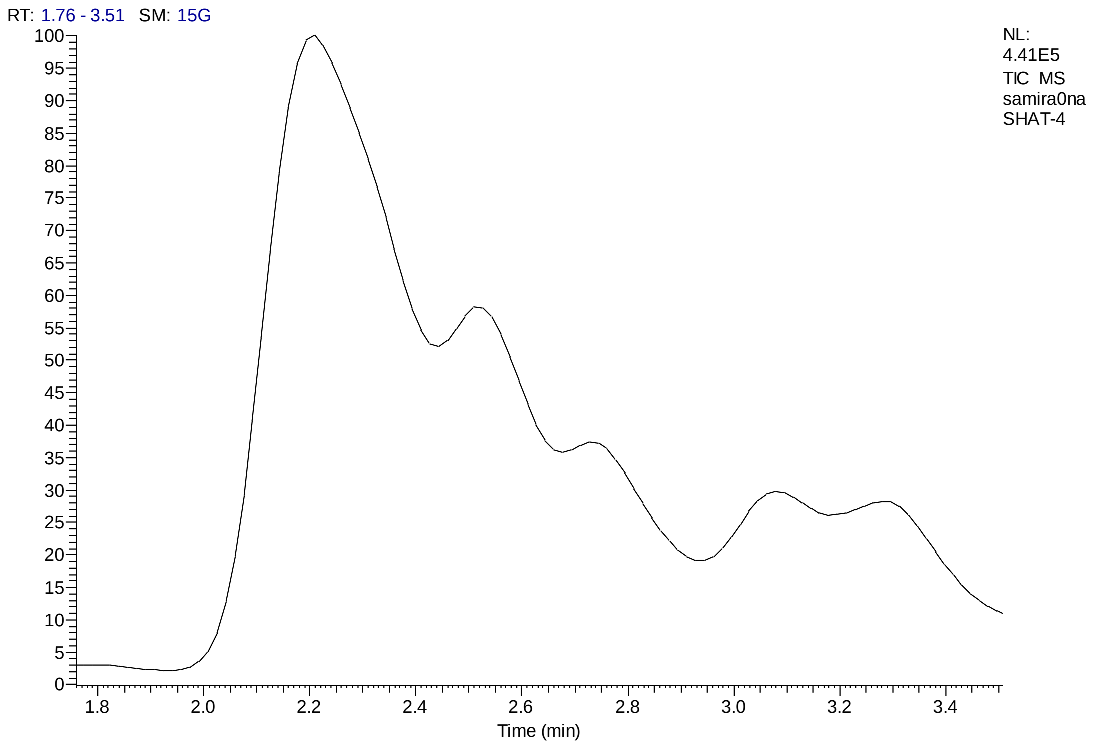

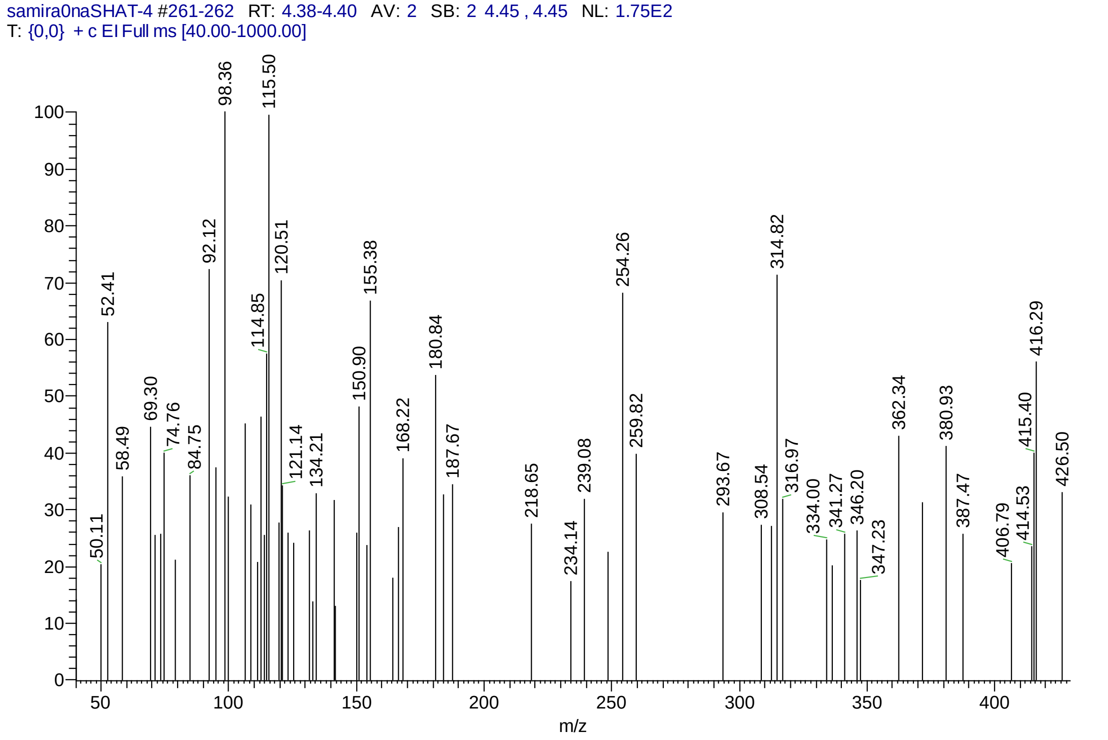

^{1}H NMR of compound 10

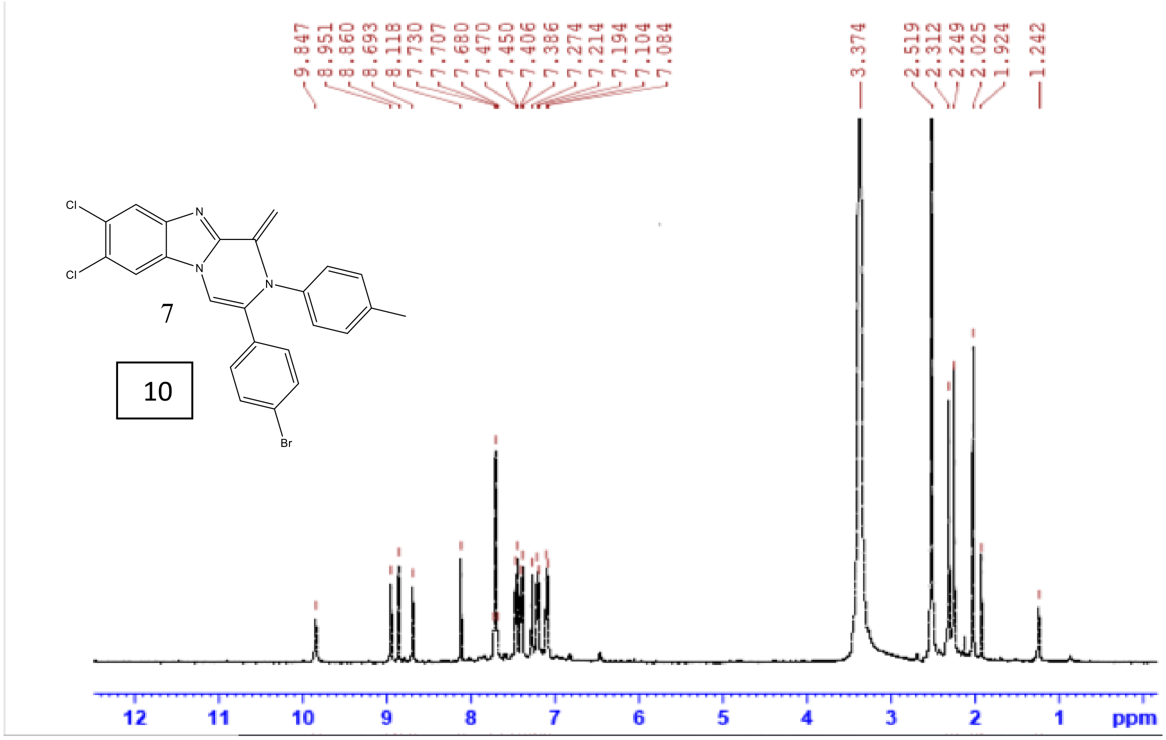

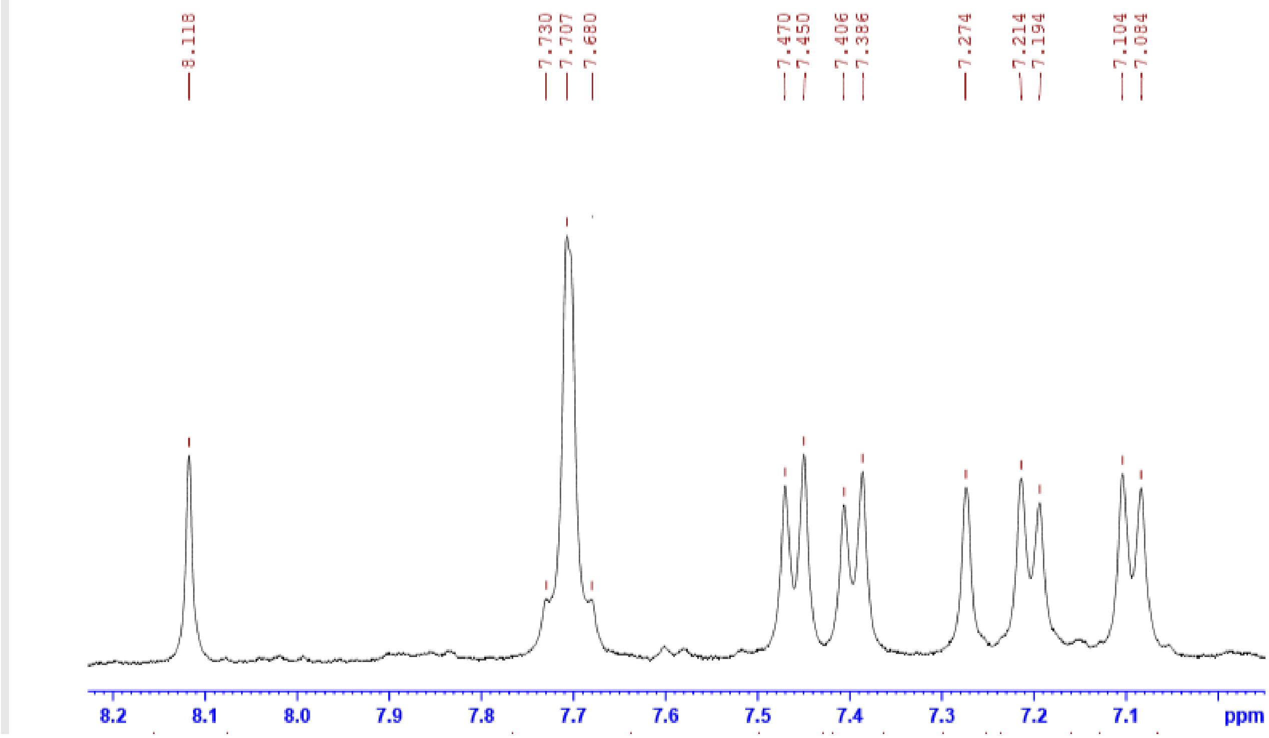

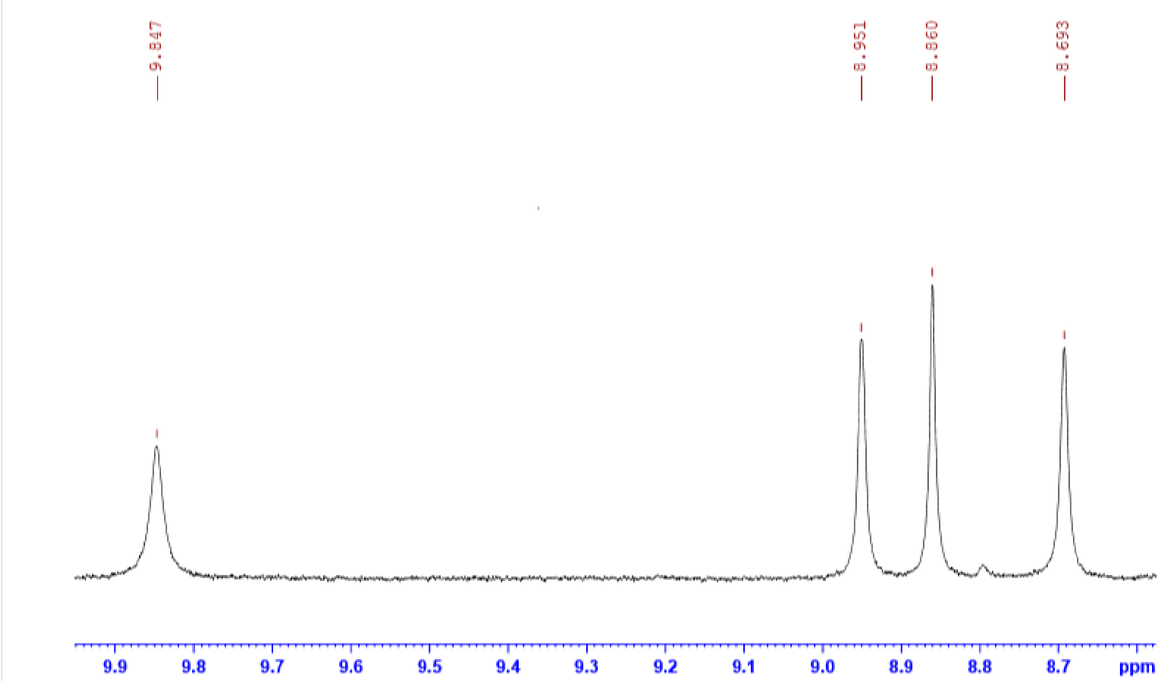

^{13}C NMR of compound 10

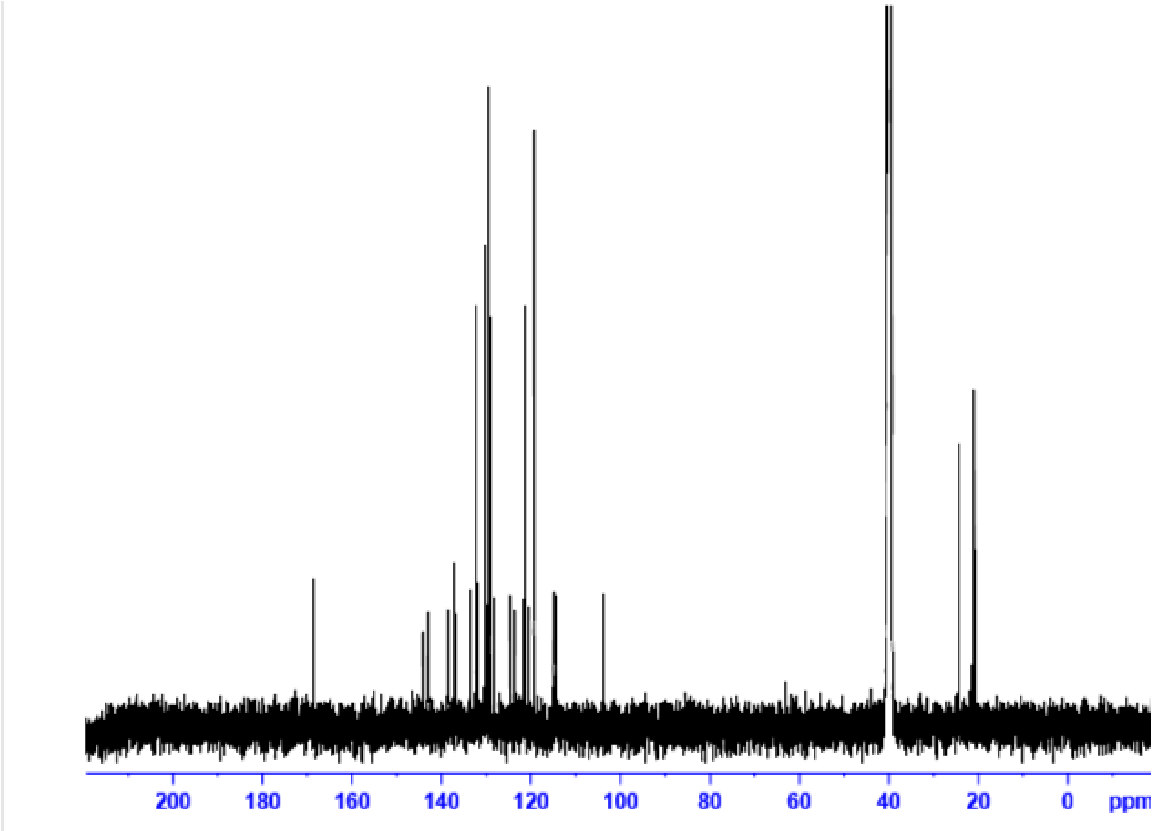

Mass of compound 10

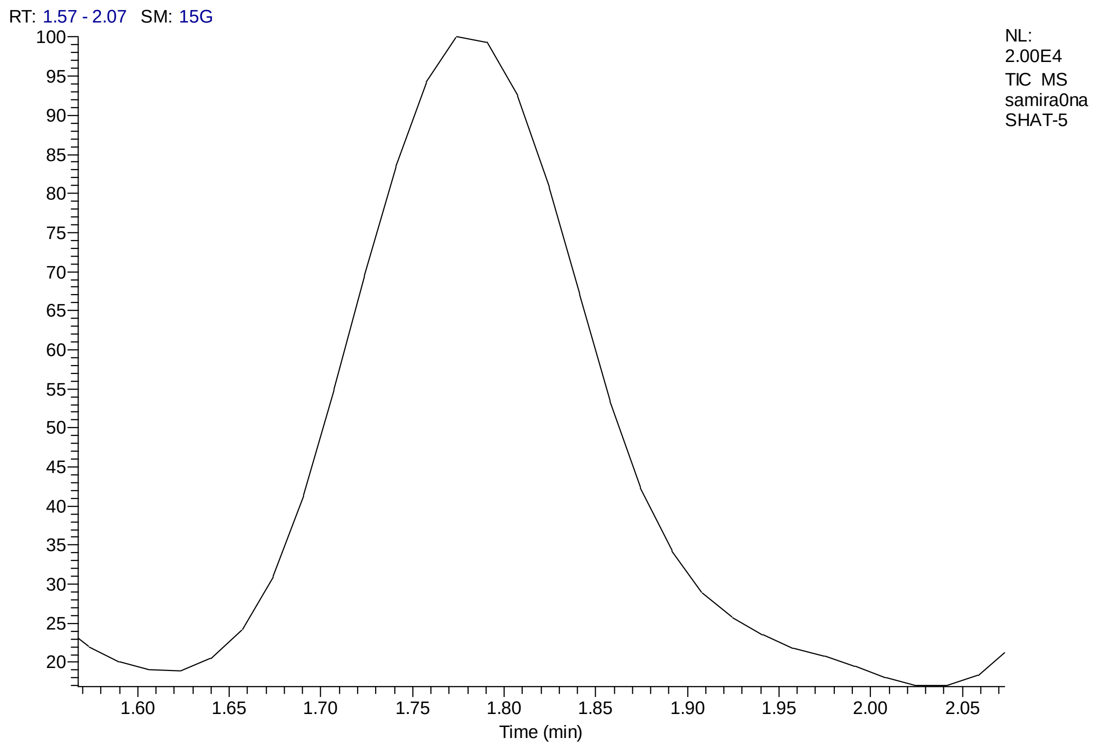

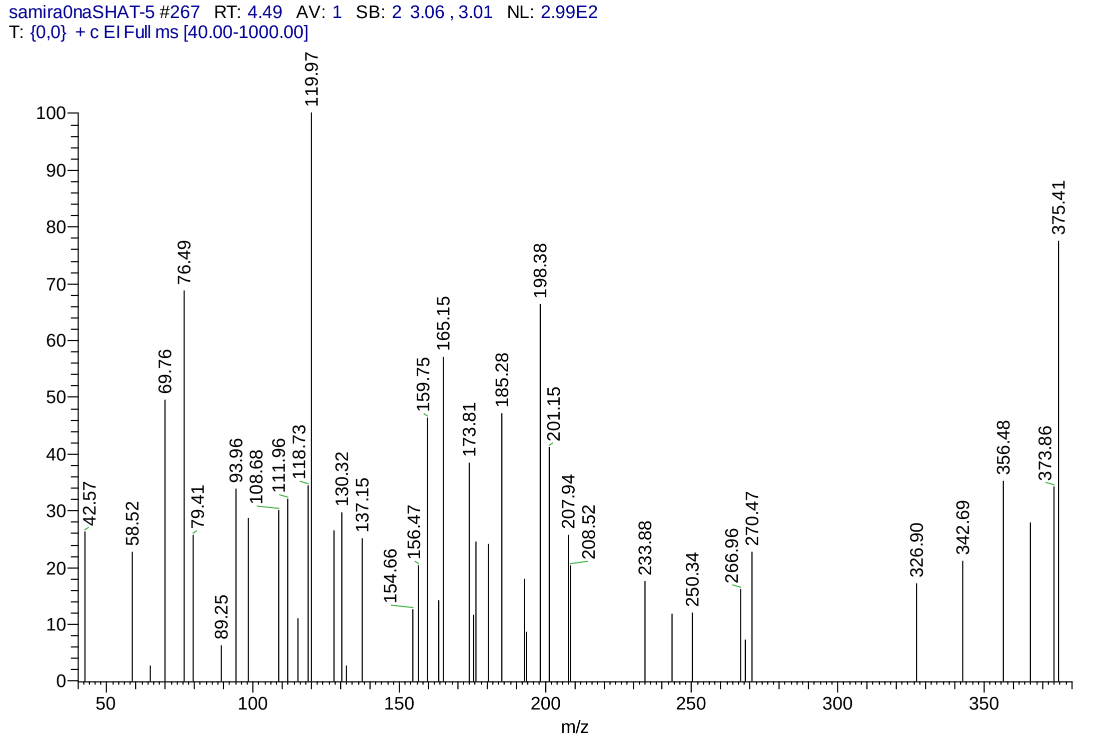

^{1}H NMR of compound 11

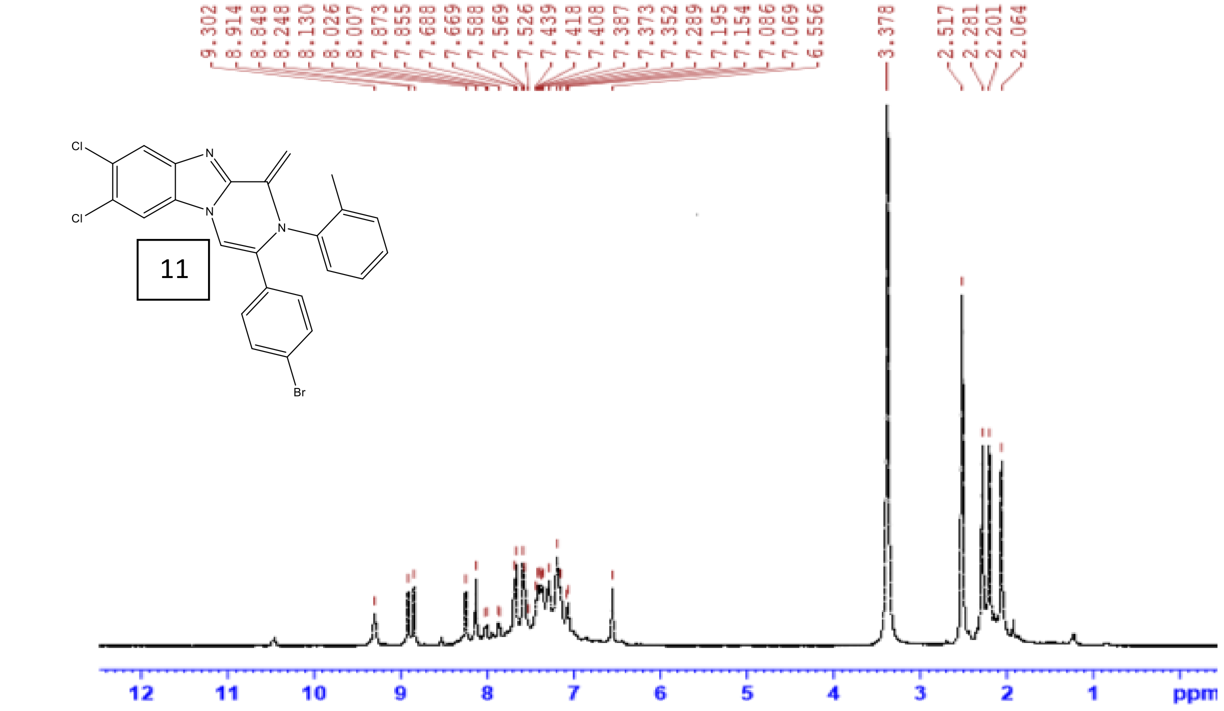

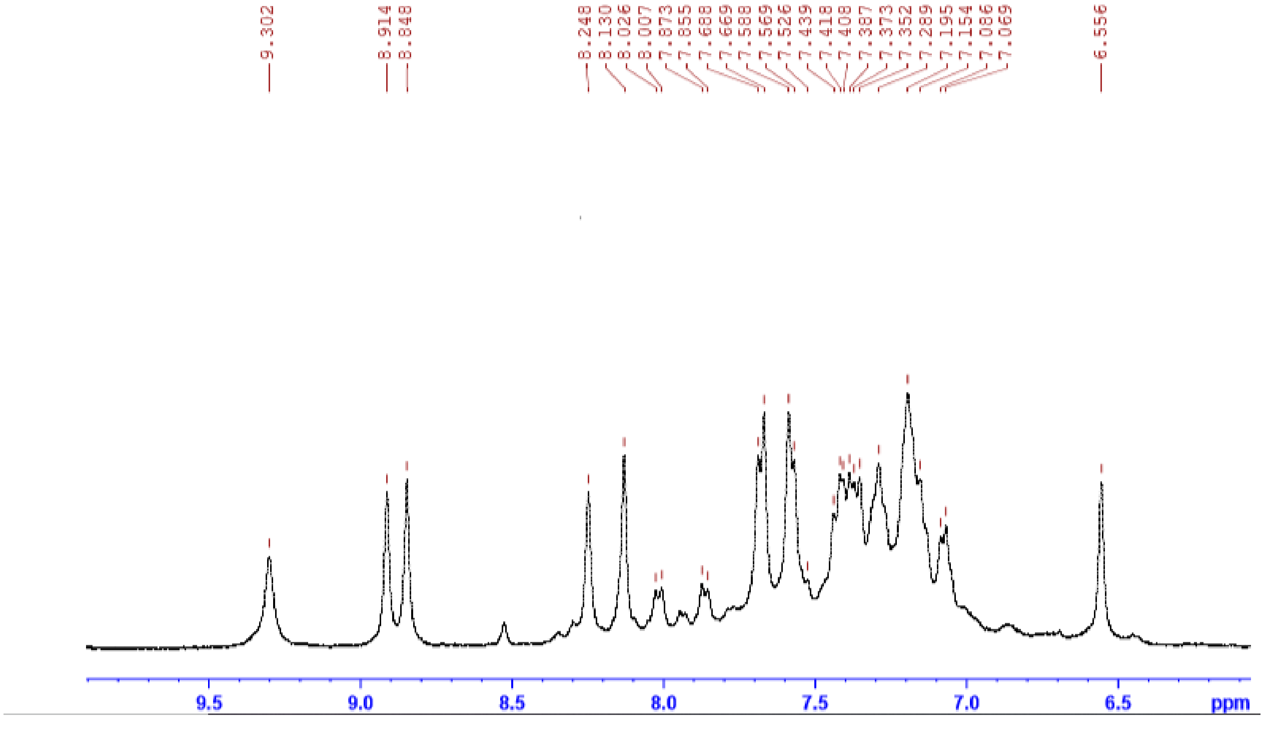

Mass of compound 11

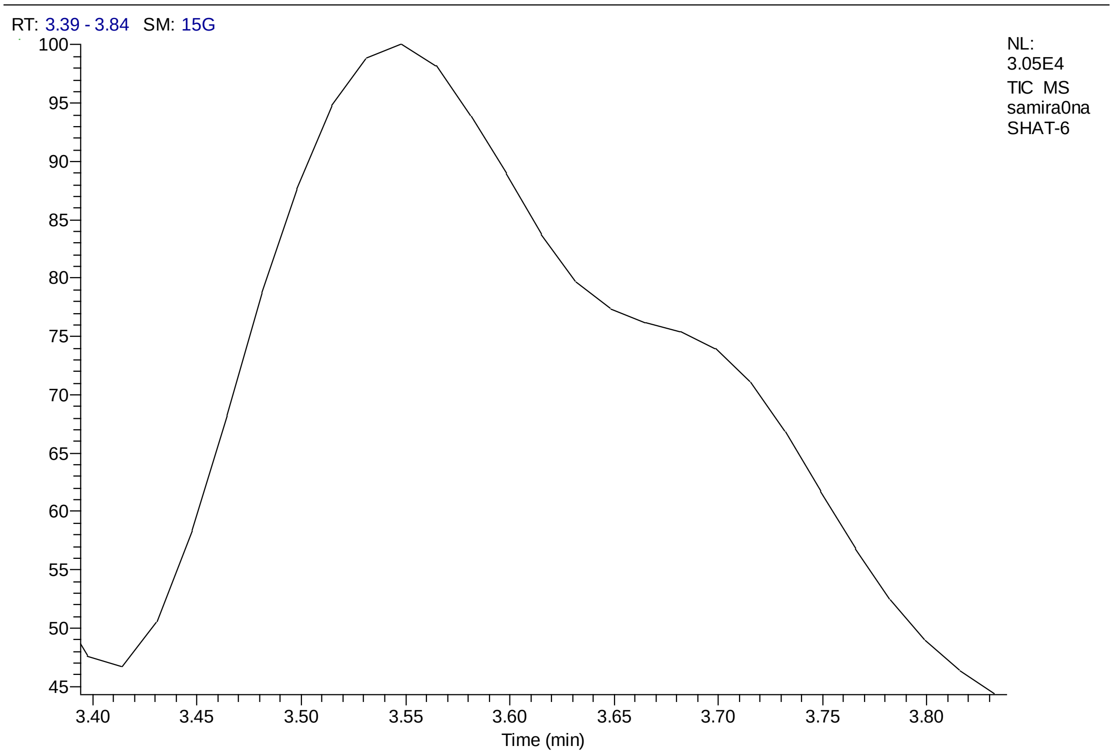

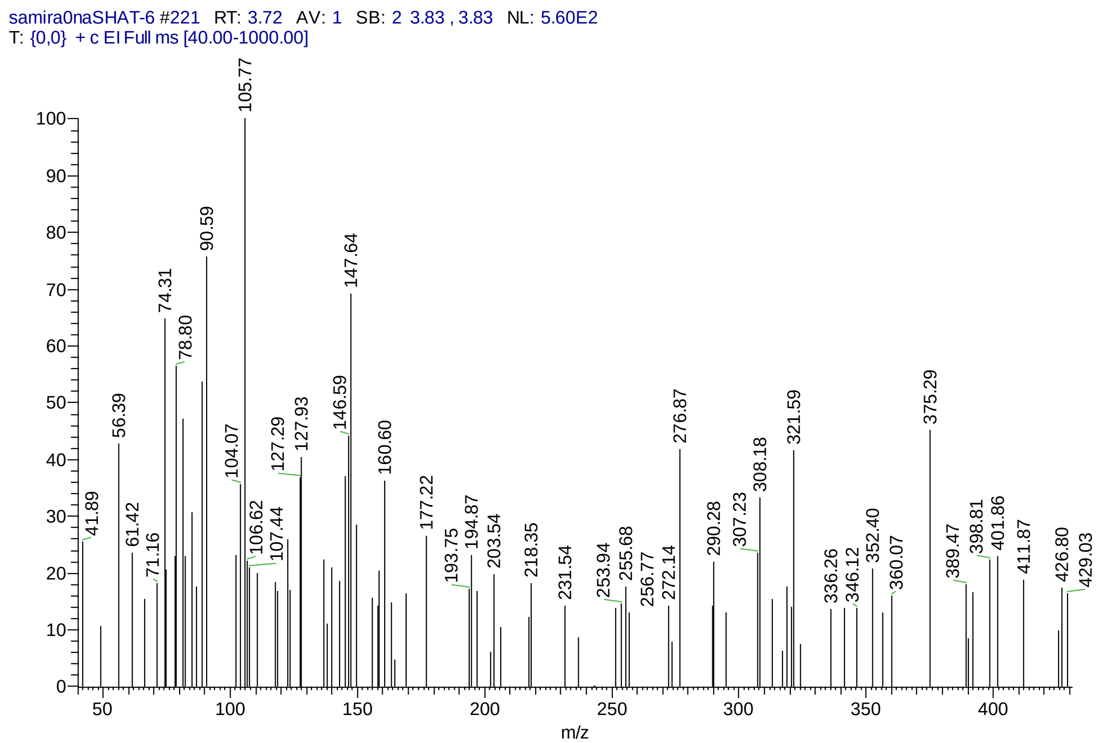

^{1}H NMR of compound 12

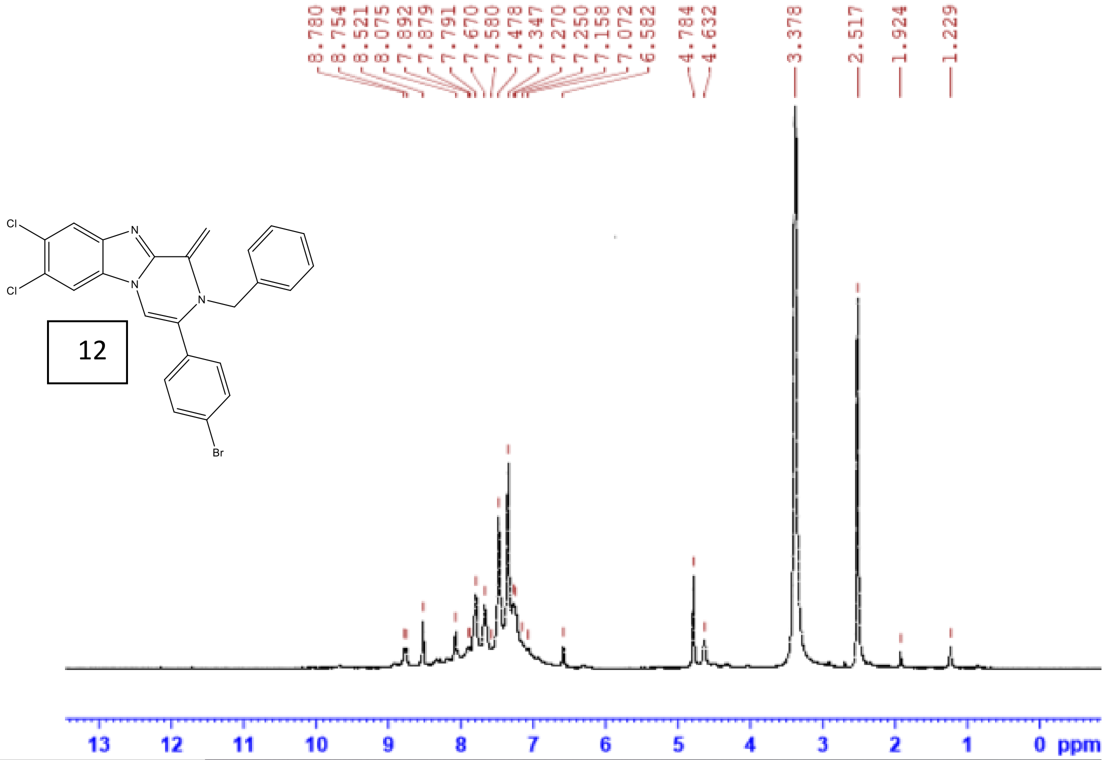
Cl
Cl
N
N
N
Br
12
8.780
8.754
8.521
8.075
7.892
7.879
7.791
7.670
7.580
7.478
7.347
7.270
7.250
7.158
7.072
6.582
4.784
4.632
3.378
2.517
1.924
1.229
13 12 11 10 9 8 7 6 5 4 3 2 1 0 ppm

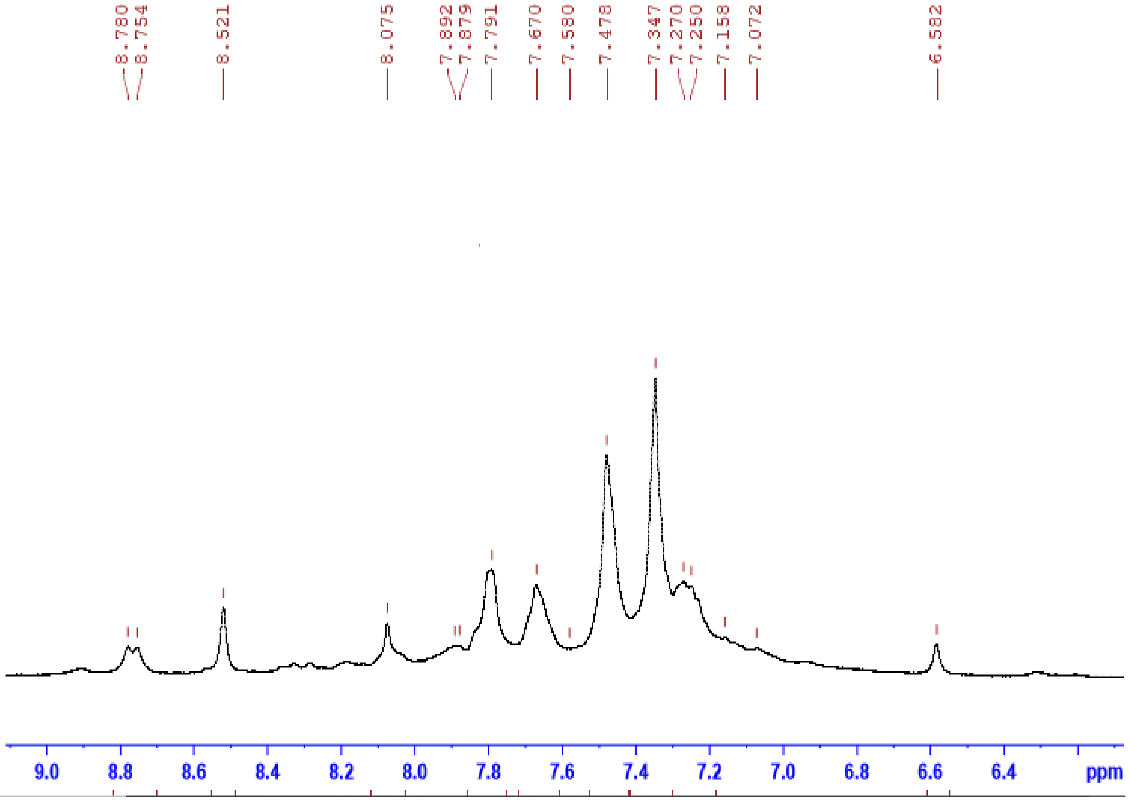
8.780
8.754
8.521
8.075
7.892
7.879
7.791
7.670
7.580
7.478
7.347
7.270
7.250
7.158
7.072
6.582
9.0 8.8 8.6 8.4 8.2 8.0 7.8 7.6 7.4 7.2 7.0 6.8 6.6 6.4 ppm

^{13}C NMR of compound 12

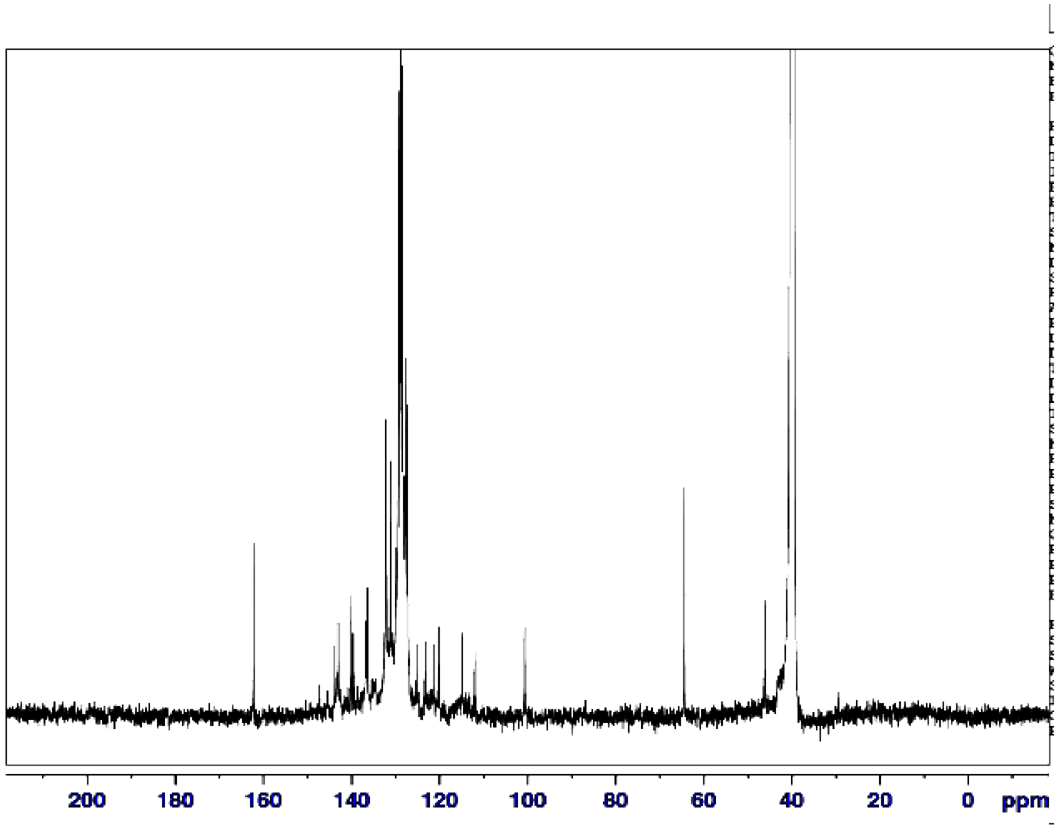

APT of compound 12

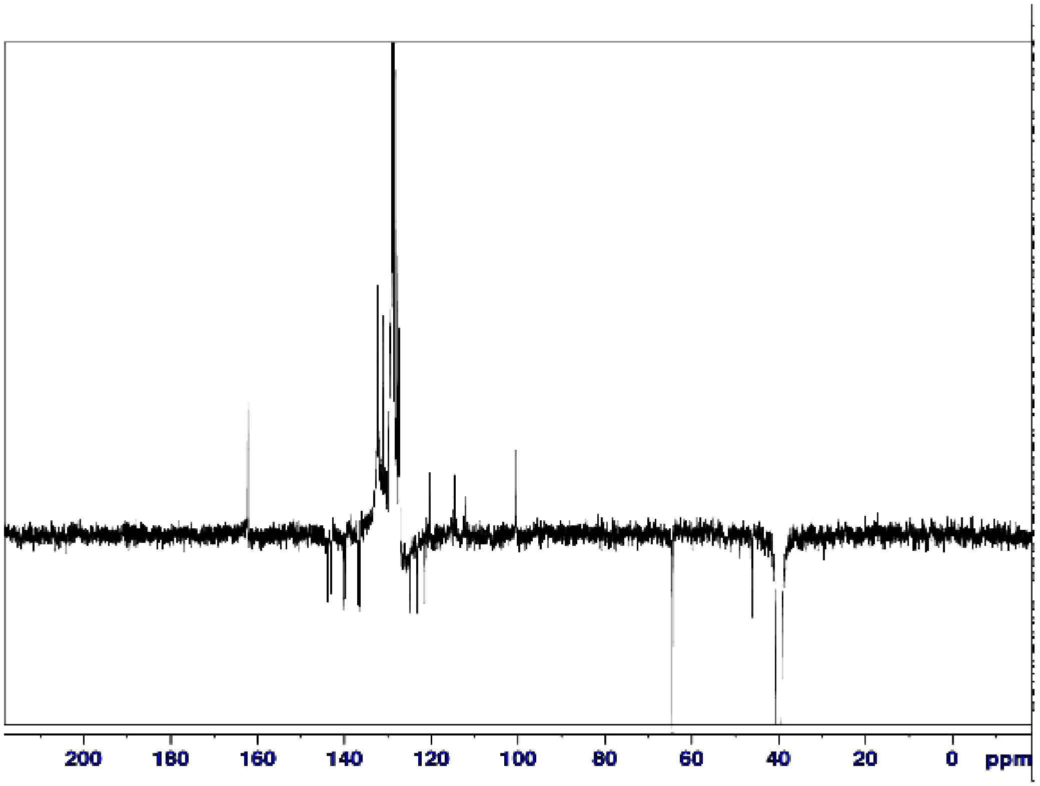

Mass of compound 12

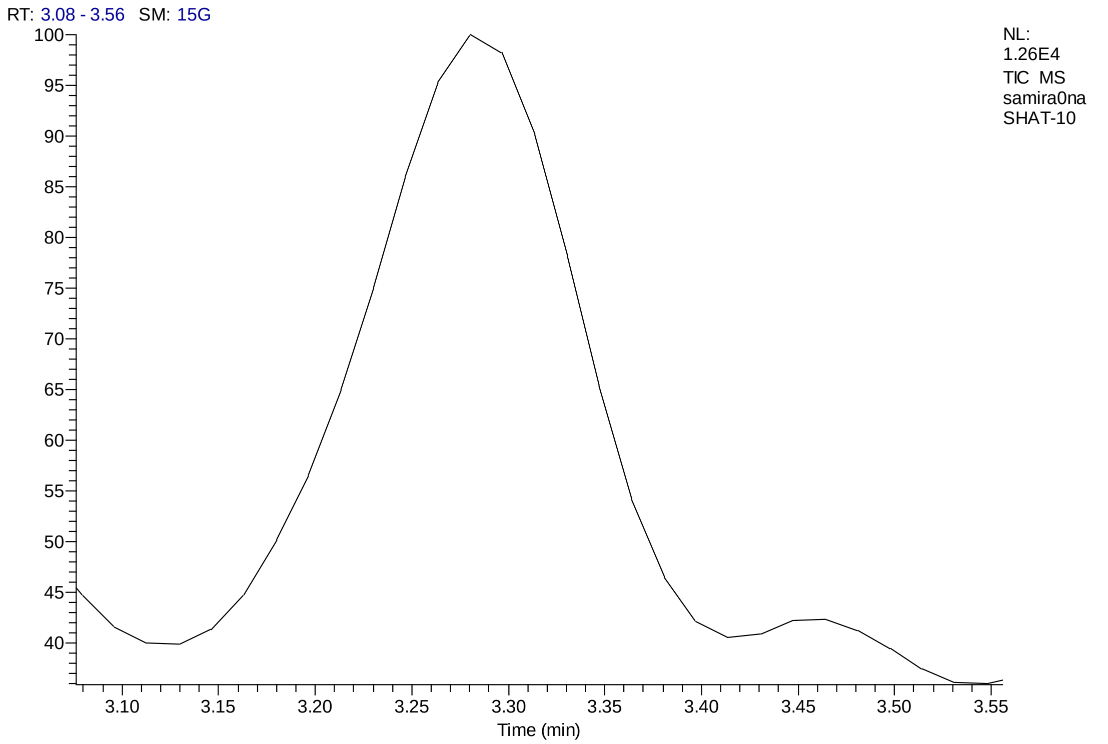

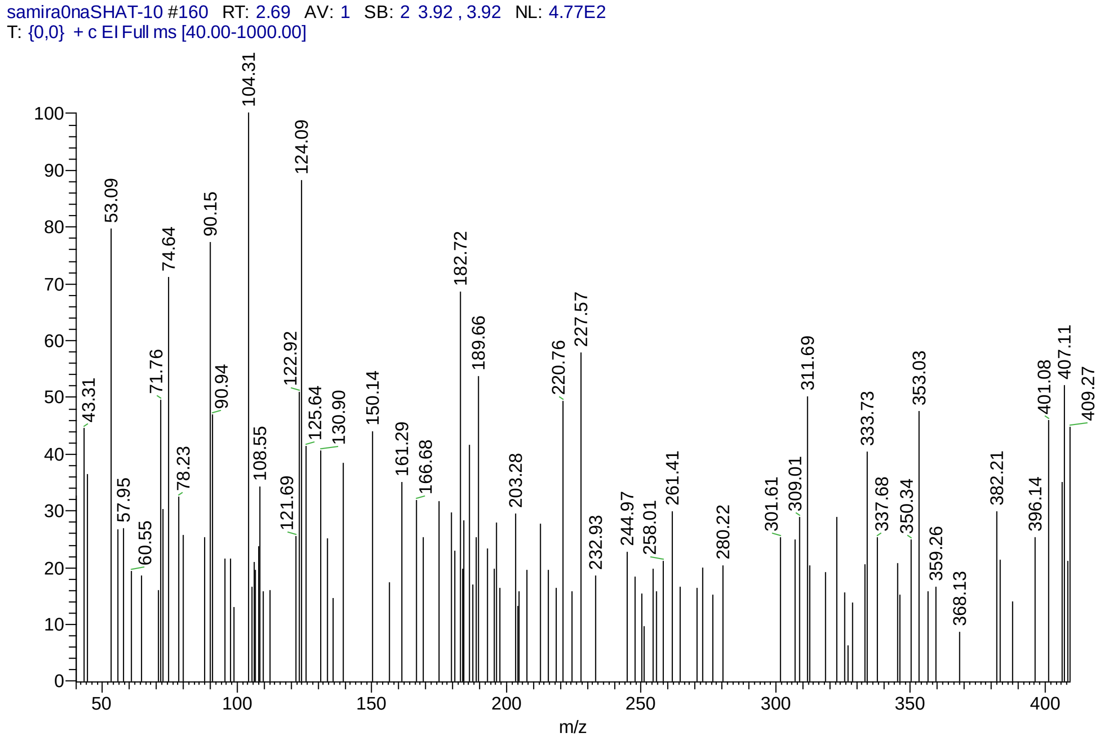

¹H NMR of compound 13

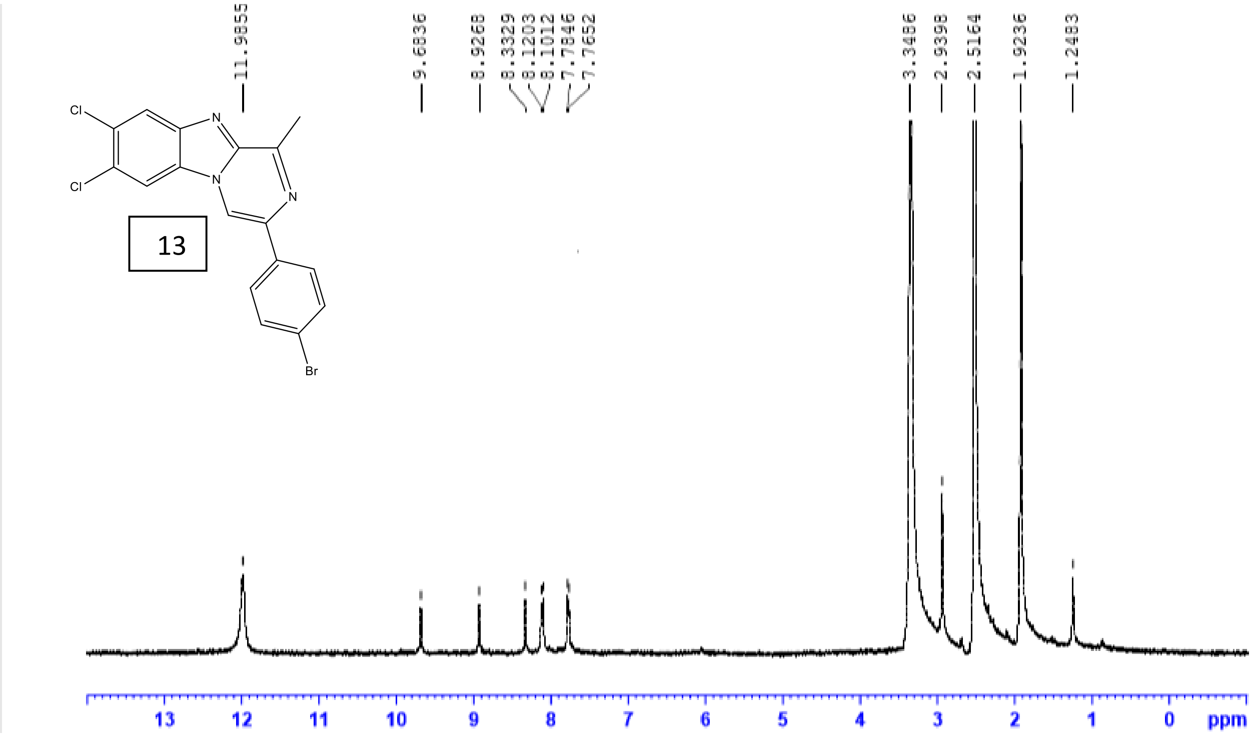

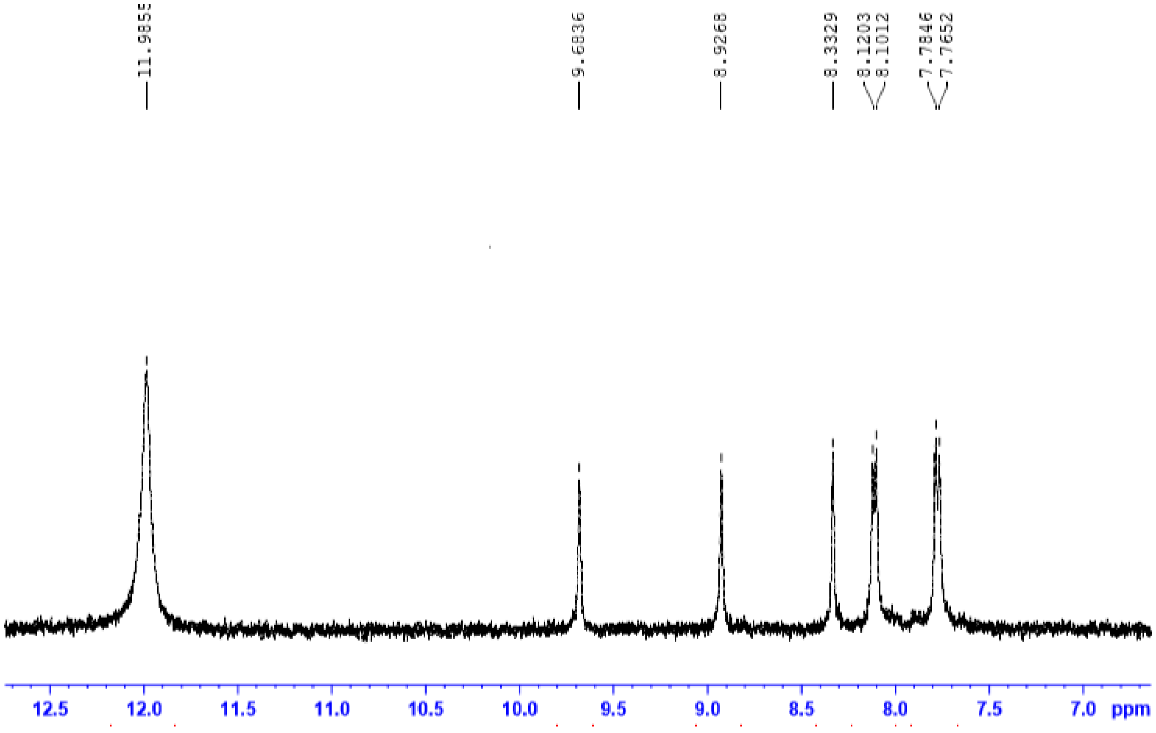

Mass of compound 13

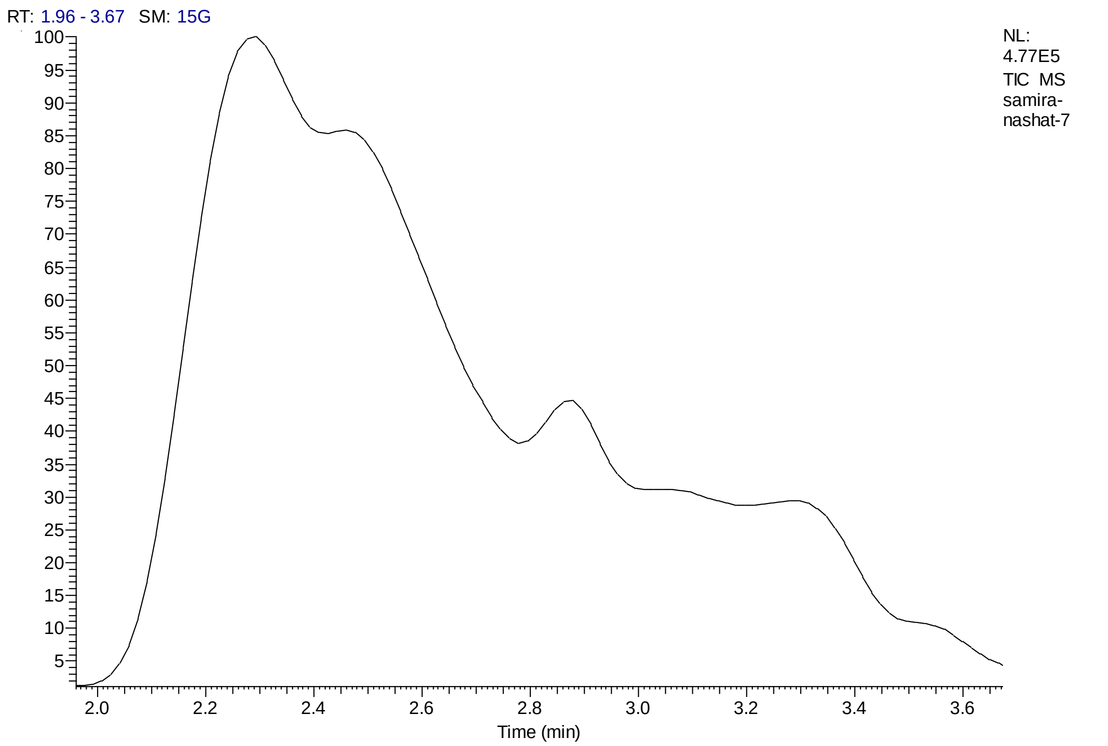

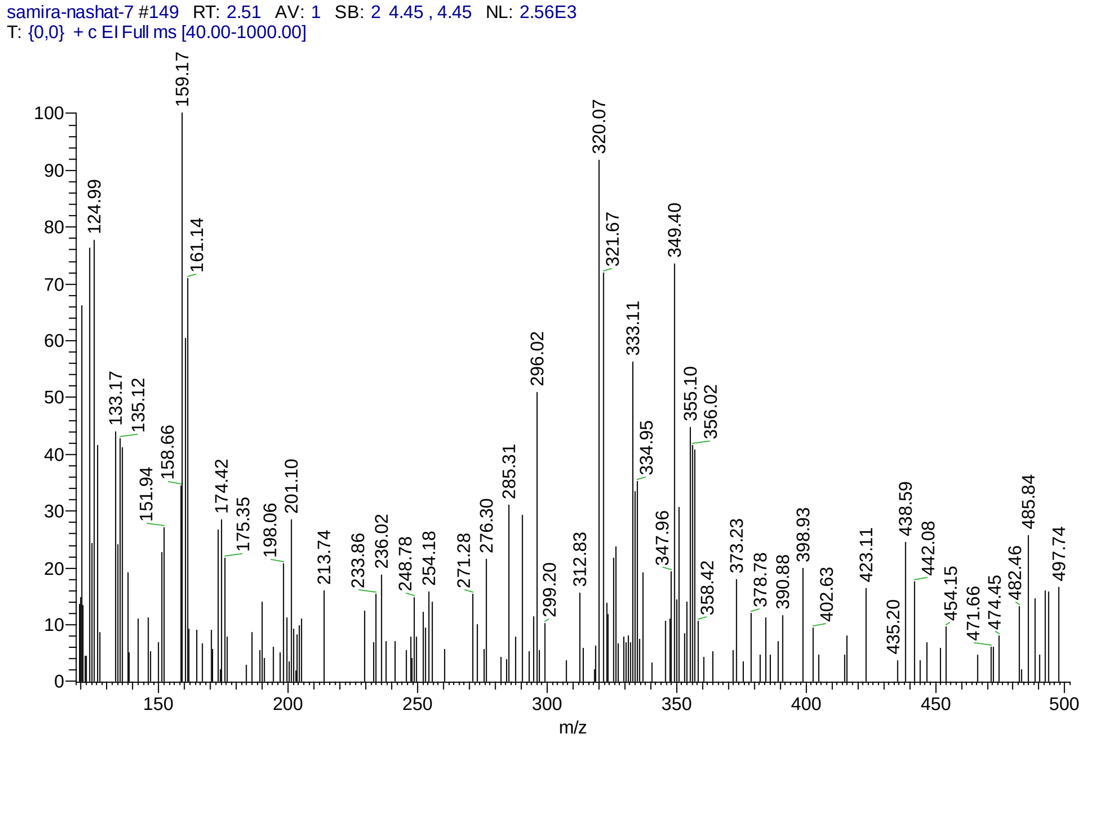

¹H NMR of compound 14

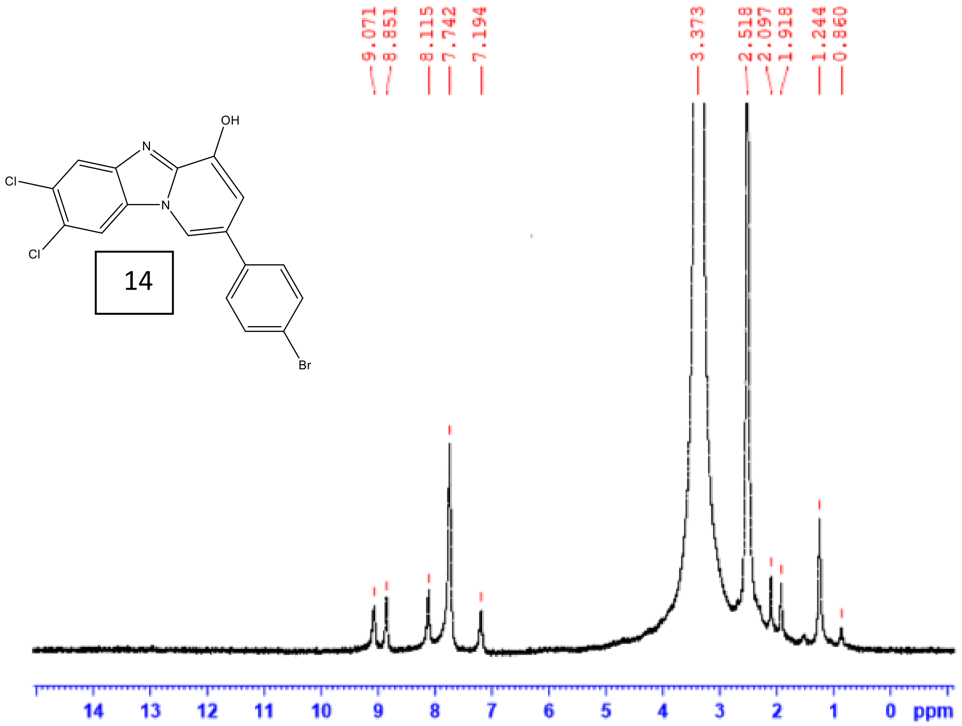

IR of compound 14

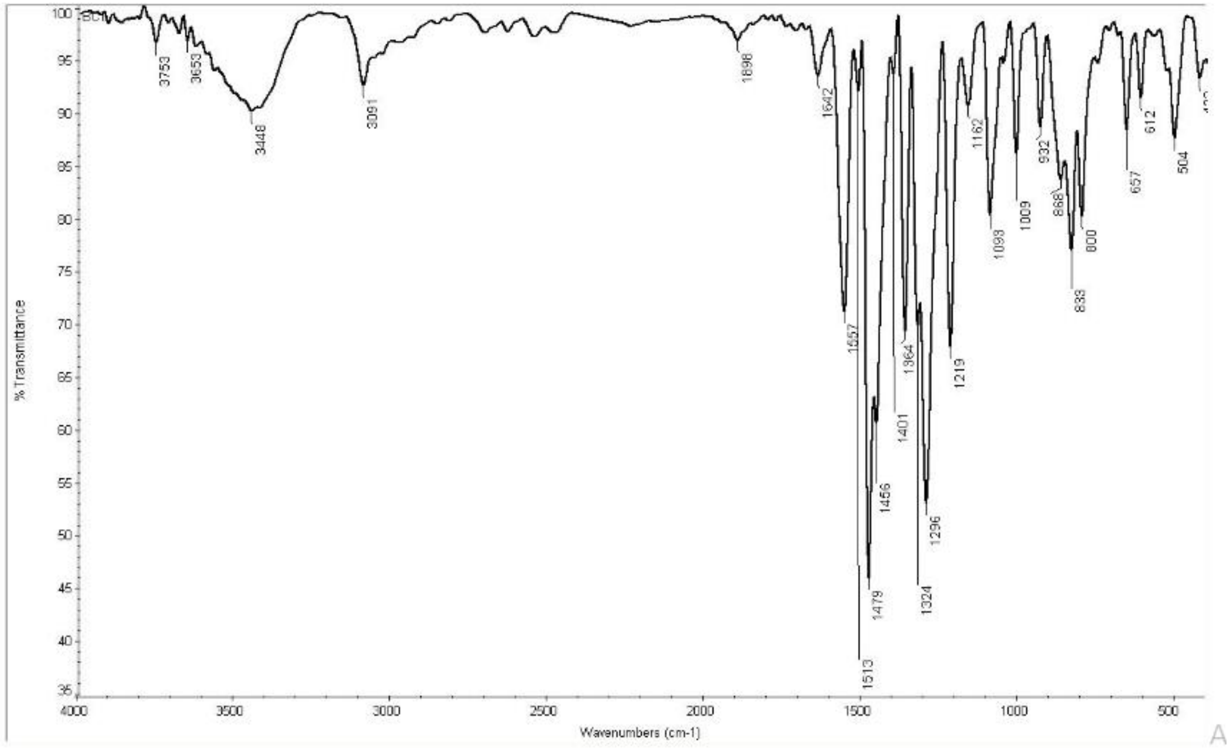

Mass of compound 14

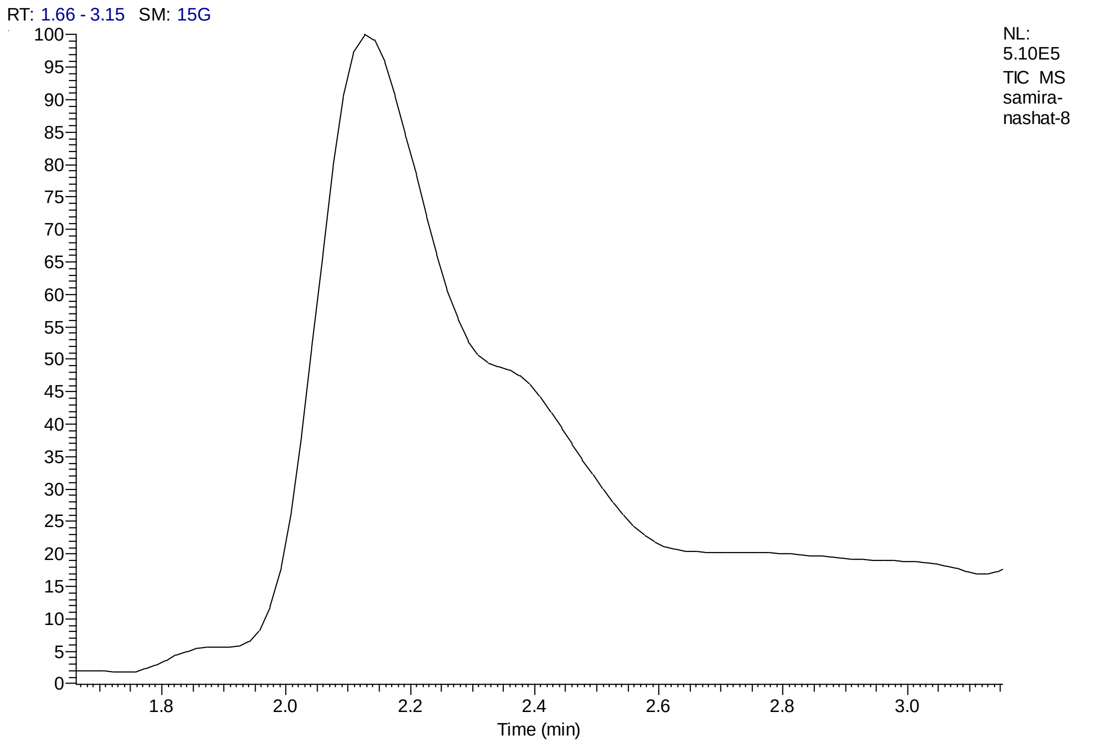

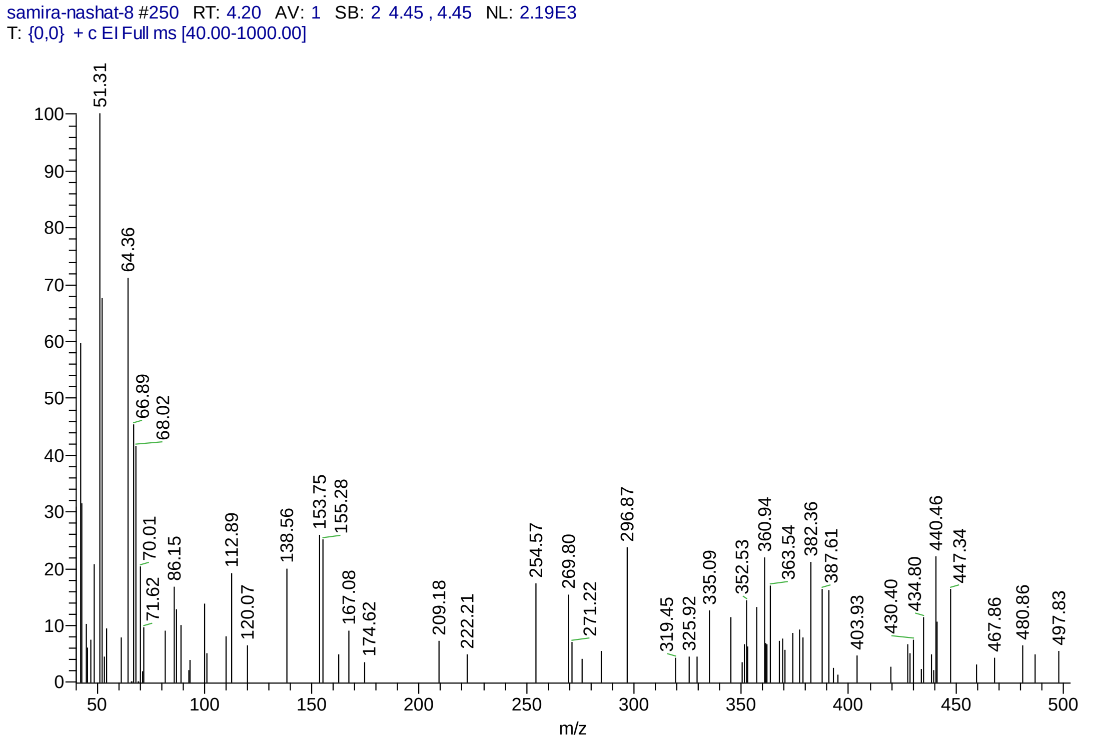

Researcher	: Dr.Samira Nashaat email: Samiranashaat2@gmail.com **mob.** 01011509651
Assay	: EGFR enzyme assay
Samples	: 05 compounds.
Cell line	: ---
Reference	: ---
Date	: 28/06/2020

<u>Lab Report</u>

**ser**	**Compound**		**Results**	
	**code**	**MW g/mol**	**pin-1 IC50 nM**	
1	2	372.25	0.646±0.03	
2	5A	362.29	2.019±0.1	
3	9	497.22	1.106±0.06	
4	15	440.62	3.609±0.16	
5	28	324.87	0.617±0.03	
6	5B	375.25	0.76±0.04	
***	Tannic acid	1701	0.535±0.02	

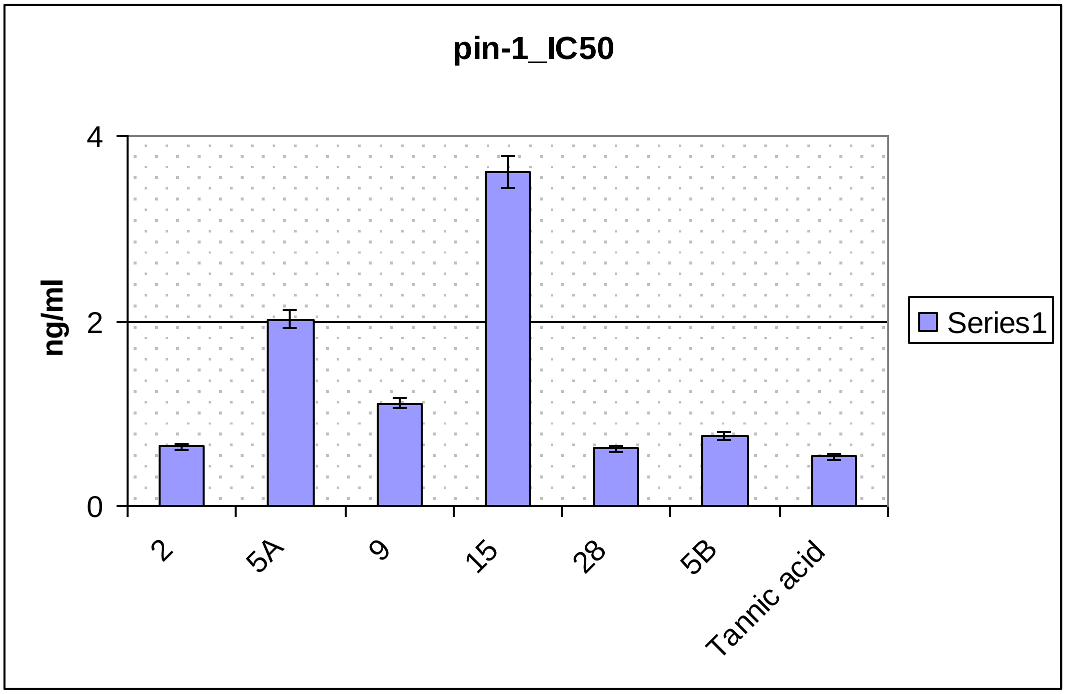
pin-1_IC50
ng/ml
4
2
0
2
5A
9
15
28
5B
Tannic acid
Series1

Detailed results

[redacted]

pin-1

[redacted]

code	IC50	conc	log	%inh	T2	T1	ΔT	RFU2	RFU1	ΔRFU	slope	K.Activity	EC
2		100	2	85.21	30	0	30	288	0	288	64.9	17.7504	120
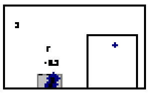		10	1	66.97	30	0	30	643	0	643	64.9	39.6302	120
		1	0	51.1	30	0	30	952	0	952	64.9	58.6749	120
		0.1	-1	39.19	30	0	30	1184	0	1184	64.9	72.9738	120
EC				0	30	0	30	1947	0	1947	64.9	120	120

[redacted]

code	IC50	conc.ng/ml	log conc	%inh	T2	T1	ΔT	RFU2	RFU1	ΔRFU	slope	K.Activity	EC
5A		100	2	84.8	30	0	30	296	0	296	64.9	18.2435	120
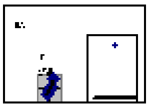		10	1	55.21	30	0	30	872	0	872	64.9	53.7442	120
		1	0	43.76	30	0	30	1095	0	1095	64.9	67.4884	120
		0.1	-1	29.94	30	0	30	1364	0	1364	64.9	84.0678	120
EC				0	30	0	30	1947	0	1947	64.9	120	120

[redacted]

code	IC50	conc.ng/ml	log conc	%inh	T2	T1	ΔT	RFU2	RFU1	ΔRFU	slope	K.Activity	EC
9		100	2	87.52	30	0	30	243	0	243	64.9	14.9769	120
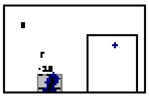		10	1	69.49	30	0	30	594	0	594	64.9	36.6102	120
		1	0	42.17	30	0	30	1126	0	1126	64.9	69.3991	120
		0.1	-1	34.72	30	0	30	1271	0	1271	64.9	78.3359	120
EC				0	30	0	30	1947	0	1947	64.9	120	120

[redacted]

code	IC50	conc.ng/ml	log conc	%inh	T2	T1	ΔT	RFU2	RFU1	ΔRFU	slope	K.Activity	EC
15		100	2	82.49	30	0	30	341	0	341	64.9	21.0169	120
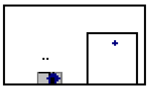		10	1	54.9	30	0	30	878	0	878	64.9	54.114	120
		1	0	34.36	30	0	30	1278	0	1278	64.9	78.7673	120
		0.1	-1	23.73	30	0	30	1485	0	1485	64.9	91.5254	120
EC				0	30	0	30	1947	0	1947	64.9	120	120

[redacted]

code	IC50	conc.ng/ml	log conc	%inh	T2	T1	ΔT	RFU2	RFU1	ΔRFU	slope	K.Activity	EC
**28**		100	2	86.7	30	0	30	259	0	259	64.9	15.963	120
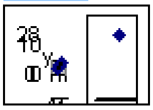		10	1	66.97	30	0	30	643	0	643	64.9	39.6302	120
		1	0	50.33	30	0	30	967	0	967	64.9	59.5994	120
		0.1	-1	40.27	30	0	30	1163	0	1163	64.9	71.6795	120
EC				0	30	0	30	1947	0	1947	64.9	120	120

[redacted]

code	IC50	conc.ng/ml	log conc	%inh	T2	T1	ΔT	RFU2	RFU1	ΔRFU	slope	K.Activity	EC
**Tannic acid**		100	2	86.03	30	0	30	272	0	272	64.9	16.7643	120
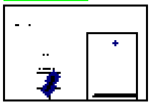		10	1	70.01	30	0	30	584	0	584	64.9	35.9938	120
		1	0	52.18	30	0	30	931	0	931	64.9	57.3806	120
		0.1	-1	39.96	30	0	30	1169	0	1169	64.9	72.0493	120
EC				0	30	0	30	1947	0	1947	64.9	120	120

[redacted]

code	IC50	conc.ng/ml	log conc	%inh	T2	T1	ΔT	RFU2	RFU1	ΔRFU	slope	K.Activity	EC
**5B**		100	2	84.8	30	0	30	296	0	296	64.9	18.2435	120
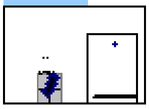		10	1	67.39	30	0	30	635	0	635	64.9	39.1371	120
		1	0	49.1	30	0	30	991	0	991	64.9	61.0786	120
		0.1	-1	38.01	30	0	30	1207	0	1207	64.9	74.3914	120
EC				0	30	0	30	1947	0	1947	64.9	120	120

**2**	y = 15.393x + 52.922
**5A**	y = 17.601x + 44.628
**9**	y = 18.572x + 49.188
**15**	y = 19.682x + 39.029
28	y = 15.593x + 53.272
**Tannic acid**	y = 15.603x + 54.242

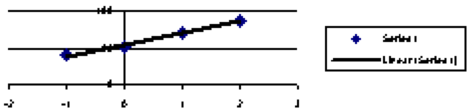

Supplement: Supplementary file 1 [file molecules-27-05245-s001.zip › molecules-1787834-supplementary.pdf]
